# Health care utilizations and costs of *Campylobacter* enteritis in Germany: A claims data analysis

**DOI:** 10.1371/journal.pone.0283865

**Published:** 2023-04-05

**Authors:** Elisabeth Schorling, Sonja Lick, Pablo Steinberg, Dagmar Adeline Brüggemann

**Affiliations:** 1 Department of Safety and Quality of Meat, Max Rubner-Institut, Federal Research Institute of Nutrition and Food, Kulmbach, Bavaria, Germany; 2 Max Rubner-Institut, Federal Research Institute of Nutrition and Food, Karlsruhe, Baden-Württemberg, Germany; Taipei Medical University, TAIWAN

## Abstract

**Objective:**

The number of reported cases of *Campylobacter* enteritis (CE) remains on a high level in many parts of the world. The aim of this study was to analyze the health care utilizations and direct and indirect costs of CE and sequelae of patients insured by a large health insurance with 26 million members in Germany.

**Methods:**

Claims data of insurants with at least one CE diagnosis in 2017 (n = 13,150) were provided, of which 9,945 were included in the analysis of health care utilizations and costs. If medical services were not diagnosis-linked, CE-associated costs were estimated in comparison to up to three healthy controls per CE patient. Indirect costs were calculated by multiplying the work incapacities by the average labor costs. Total costs of CE in Germany were extrapolated by including all officially reported CE cases in 2017 using Monte Carlo simulations.

**Results:**

Insurants showed a lower rate of 56 CE diagnoses per 100,000 than German surveillance data for 2017, but with a similar age, gender and regional distribution. Of those CE cases, 6.3% developed post-infectious reactive arthritis, Guillain-Barré syndrome (GBS), inflammatory bowel disease (IBD) and/or irritable bowel syndrome (IBS). Health care utilizations differed depending on CE severity, age and gender. Average CE-specific costs per patient receiving outpatient care were € 524 (95% CI 495–560) over a 12-month period, whereas costs per hospitalized CE case amounted to € 2,830 (2,769–2,905). The analyzed partial costs of sequelae ranged between € 221 (IBS) and € 22,721 (GBS) per patient per 12 months. Total costs of CE and sequelae extrapolated to Germany 2017 ranged between € 74.25 and € 95.19 million, of which 10–30% were due to sequelae.

**Conclusion:**

CE is associated with a substantial economic burden in Germany, also due to care-intensive long-lasting sequelae. However, uncertainties remain as to the causal relationship of IBD and IBS after CE.

## Introduction

*Campylobacter* is a gram-negative bacterium that can cause acute gastrointestinal infections in humans. In the European Union, *Campylobacter* enteritis (CE) is the most reported gastrointestinal disease and the number of confirmed CE cases has stabilized on a high level in recent years [1]. The corresponding incidence or notification rate in the EU-27 was 65 cases per 100,000 inhabitants in 2017. In Germany, CE is the most common notifiable bacterial infection and the incidence rate in 2017 was 84 reported cases per 100,000 inhabitants according to national surveillance data [1–3].

The symptoms associated with CE encompass diarrhea, abdominal pain, fever, nausea, vomiting and/or bloody stools, and are generally self-limiting within two weeks [3, 4]. However, severe courses of disease can lead to hospitalizations and different long-term complications weeks or even years after an infection. Among reported post-infectious sequelae of CE are reactive arthritis (REA), Guillain-Barré syndrome (GBS), inflammatory bowel disease (IBD) and irritable bowel syndrome (IBS) [5–7].

Risk factors for CE include the handling, preparation and consumption of chicken meat, drinking unpasteurized milk or untreated water, eating out, traveling, use of proton pump inhibitors (PPI) and animal contact, especially with poultry [4, 8]. Various control options and prevention strategies are discussed, many of which are aimed at reducing *Campylobacter* contaminations in the broiler food chain [9]. However, for a reliable evaluation of benefits and costs associated with possible interventions in the long term, it is crucial to use country-specific data on the current health and economic burden.

Several disease models were established to estimate the health and/or economic burden associated with CE and sequelae in different countries, and two of them were developed for Germany [10, 11]. So far, there was only one estimation of the health burden of CE including sequelae measured in disability adjusted life years (DALY) for Germany [11], but no evaluation of the cost of illness (COI).

In recent years, COI studies for CE were performed in Europe (i.e. in the Netherlands, Sweden, Switzerland and United Kingdom), New Zealand and North America, e.g. [12–17]. They show that a major part of the economic burden of CE is due to costly sequelae and an estimated high number of unreported cases of CE. However, health care utilizations and costs in different countries are difficult to compare due to differences in the reimbursement systems and system-specific care pathways [18, 19].

In this study, we present an overview of the health care utilizations and associated costs of CE and sequelae in Germany over a 12-month period, which were analyzed based on the claims data of a large health insurance representing 26 million members. The study was designed and conducted by taking into consideration the recently published checklist for COI studies in the German context [20]. In addition, the rates of diagnosed CE among insurants were compared with the officially reported incidence rates according to German surveillance data, and the development of post-infectious sequelae was evaluated over a period of up to three years.

## Methods

### Data and study population

Anonymized claims data of a statutory health insurance (SHI) in Germany were used to assess the CE-specific health care utilizations and costs. The SHI in Germany is characterized by an universal health coverage with comprehensive benefits [21]. The AOK Research Institute (WIdO) has access to health care utilization and reimbursement data of 26 million people insured by AOK in Germany [22]. All data were fully anonymized by WIdO; therefore, an approval of an ethics committee was not required.

Data sets of insurants with at least one diagnosis of CE (code A04.5 according to the International Classification of Diseases (ICD), 10^th^ revision, German Modification [23]) between the 1^st^ of January 2017 and the 31^st^ of December 2017 were provided by WIdO. However, the exact diagnosis date is usually not recorded within the claims data. Thus, insurants with CE were included either on the basis of the date of an outpatient CE-associated benefit, where available; otherwise, the first day of the regular quarterly diagnosis reports was used. In case of hospitalized insurants, the hospital discharge dates were taken (hospitalizations that did not end until 2018 were also included). In this study, CE-associated claims were defined according to the positions of the doctor’s fee scale within the SHI scheme [24] either as i) specific laboratory tests (position 32588: qualitative and/or quantitative proof of antibodies against *Campylobacter*, position 32723: stool test with at least five cultural media including testing for *Yersinia (enterocolitica)* and *Campylobacter spp*., position 32707: detection of bacterial antigens, and/or position 32001: provision or arrangement of laboratory services in general) or ii) mere medical suspicion of a reportable disease (position 32006). Subsequent CE diagnoses were considered as separate (new) CE diagnoses, if the timespan between the two diagnoses exceeded 90 days [25]. In the current study, only confirmed outpatient and hospital-made CE diagnoses were included. For the evaluation of diagnosis frequency, however, suspected outpatient diagnoses were also considered, whereas in a separate sensitivity analysis this was not the case.

The individual study period started with the quarter of the first CE diagnosis in 2017 and lasted until the 31^st^ of December 2019. For a few hospitalized patients, the study period started with the quarter of their discharge from hospital in 2018. Insurants who were not continuously insured throughout the study period were excluded from the analysis. This also applies to insurants, which had passed away.

The data sets comprised detailed information about health care utilizations and costs covered by the health insurance as well as co-payments made by insurants themselves referring to i) outpatient medical care, ii) inpatient medical care, iii) prescribed medication and iv) inpatient rehabilitation measures. For most medical services, details regarding the underlying diagnoses were available. Additionally, notifications and duration of incapacities for work as well as sociodemographic characteristics of insurants (year of birth, gender, German Federal State affiliation) could be obtained. Insurants were divided into six age groups: <5, 5 to 14, 15 to 29, 30 to 44, 45 to 64 and ≥65 years in consideration of i) the reported CE incidence rates, which are highest among small children and young adults [2, 3] and ii) the average working age of 15 to 64 years [26].

Disease severity was categorized based on the health care utilizations in the first year of the individual study period into:

- moderate: insurants seeking outpatient medical care and/or inpatient medical care with a secondary CE diagnosis, or
- severe: insurants seeking inpatient medical care with a principal CE diagnosis.

Insurants with asymptomatic CE or with mild CE symptoms, which had not made use of any (claimable) medical services, were not considered in the claims data analysis.

**Fig 1:**
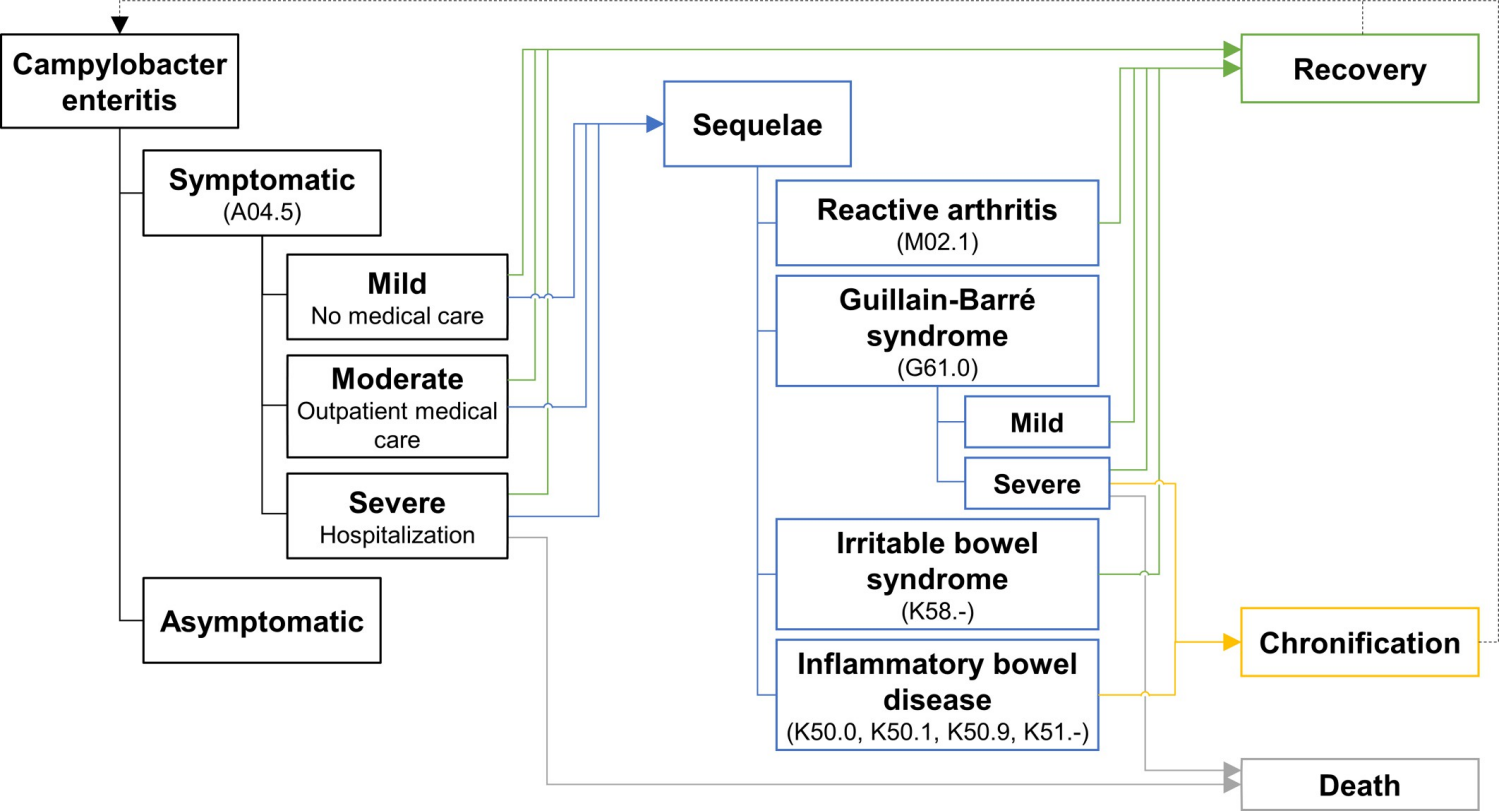
Disease model of *Campylobacter* enteritis and sequelae. Disease model based on established models [10, 11]. ICD-10 codes are shown in parentheses.

In addition, probable sequelae of CE were also evaluated. Diseases as listed below were considered to be CE-associated in accordance with other established disease models of campylobacteriosis [10, 11]:

- reactive arthritis (REA), ICD-10 code M02.1,
- Guillain-Barré syndrome (GBS), ICD-10 code G61.0,
- inflammatory bowel disease (IBD): Crohn’s disease (CD) and ulcerative colitis (UC), ICD-10 codes K50.0, K50.1, K50.9 and K51.-,
- irritable bowel syndrome (IBS), ICD-10 code K58.-,

provided that these diagnoses were made concurrently with or subsequent to a CE diagnosis. Preexisting REA, GBS, IBD or IBS were not considered as sequelae, if they were already diagnosed within 12 months prior to the individual study period. The disease model is displayed in Fig 1.

### Control group

In order to compare insurants with and without CE regarding i) the occurrence of sequelae, ii) the presence of comorbidities and iii) the costs of medical services not diagnosis-linked, data were also provided for a control group: For each insurant with at least one CE diagnosis in 2017 (*patient*), three randomly selected insurants (*control*) matching in terms of age, gender and place of residence (according to German Federal State affiliation) were included. Controls were required to have no history of campylobacteriosis, i.e. no CE diagnoses within 12 months prior to and during the study period, and they had to be continuously insured. The beginning of the individual study period in controls was set to be identical to that of the matched patients.

Existing comorbidities in patients and controls during the first year of the study period were assessed by two indices: Firstly, the Elixhauser comorbidity index of 30 comorbidities, which were originally evaluated based on ICD-9 codes [27]. The adaption to ICD-10-GM was performed according to the coding algorithm by Quan et al. [28]. Secondly, the pharmacy-based metric with the ATC algorithm [29], a classification into 32 chronic conditions according to prescription claims data that allowed to identify comorbidities, which might not have been recorded with an ICD code. Since there were significant differences in comorbidities between patients and controls, a subgroup was formed in a second step, for which patients and controls were additionally matched by comorbidities according to either the Elixhauser comorbidity index or the pharmacy-based metric (with the exception of *pain* according to the pharmacy-based metric, as pain killers can be essential in the treatment of CE [30]). In order to avoid bias due to differences in the existing comorbidities, CE-associated costs of medical services not linked to diagnoses were estimated in this subgroup by an excess cost approach as described below.

### Health care utilizations and work incapacities

All analyses were performed from a societal perspective [18, 19]. The utilization of the following medical services directly associated with CE or sequelae was analyzed:

- outpatient medical care: consultations of general practitioners (GP) and specialists associated with (confirmed) CE diagnoses or (confirmed or suspected) diagnoses of sequelae or consultations provided in hospitals,
- inpatient medical care: hospital stays associated with principal diagnoses of CE or sequelae,
- inpatient rehabilitations: rehabilitations of non-working patients associated with diagnoses of sequelae.

Additionally, to determine productivity losses, the frequency and average duration of disease-related temporary work incapacities were assessed. The proportion of patients that had received prescriptions for medicines were also evaluated. As those prescriptions were not linked to diagnoses, the information given in national medical treatment guidelines was used instead. Medication for the treatment of CE recommended in the above-mentioned guidelines include antibiotics such as azithromycin or ciprofloxacin (but not erythromycin), analgesics and spasmolytics as paracetamol, opioids, metamizole and/or butylscopolamine, and oral rehydration solutions in special severe cases, when children are involved [30, 31]. A prescribed medication (classified according to the German version of the ATC system [32]) was considered CE-associated, if it occurred within the same time frame (quarterly settlements) as the CE diagnosis.

The possibility that the use of PPI such as omeprazole and pantoprazole may increase the susceptibility to enteric diseases including CE is under discussion [33]. Therefore, the proportion of patients and controls having been prescribed PPI (ATC code A02BC [32]) within four weeks prior to the individual study period according to the pharmacy dispensing date was determined.

### Cost of illness

The costs of *Campylobacter* enteritis and sequelae were calculated by analyzing direct and indirect costs [18, 19]. The direct health care costs correspond to the payments by SHI and the co-payments by patients according to the claims data. Regarding the costs of prescribed medication, discounts to be granted by pharmaceutical companies and pharmacies (according to §§ 130 and 130a SGB V) have been taken into account. In order to assess indirect costs due to productivity losses, average labor costs were used to monetize each day of work incapacity: The average labor costs in Germany are reported per working hour and were € 34.20 in 2017 [34]. Therefore, the labor costs per working day were estimated by assuming that the average working time of 39.4 and 30.8 hours for men and women aged 15 to 64 years, respectively [35], was distributed over five days a week. The resulting labor costs per working day were € 269 for men and € 211 for women.

The costs of medical care in hospitals, of rehabilitations and of work incapacities linked to diagnoses of CE or sequelae were assessed separately for each patient. The costs of outpatient medical care and of prescribed medication are usually not directly diagnosis-linked in the claims data. Therefore, CE-specific costs were estimated as additional costs in comparison to basic costs as incurred in the respective controls in the first year of the study period via two-part regression models: First, logistic regression was used to fit the probability of non-zero costs in moderate and severe CE. Then, the costs were estimated using an ordinary least squares regression of log-transformed non-zero costs. Age group and gender were included as explanatory variables. Regression analyses were performed in the subgroup of patients and controls matched by comorbidities. A similar estimation of sequelae-specific costs of outpatient medical care and of prescribed medication was not performed due to small case numbers.

### Extrapolation of total costs in Germany

Total costs of CE cases in Germany in 2017 were extrapolated following an incidence approach: Based on the costs obtained over 12 months, total costs of all 2017 officially reported CE cases in Germany according to surveillance data [2] were calculated. These also include the costs due to sequelae in subsequent years. For this purpose, some assumptions had to be made and additional data sources were used (a complete listing of all parameters is provided in Table H in S1 File):

- Average patient groups with increasing age range were formed and additionally separated according to gender (see above). However, in order to reflect differences in case numbers, mortality and the remaining life expectancy of the elderly, the age group ≥65 years was subdivided into two groups (65 to 74 and ≥75 years).
- For each patient group, age- and gender-specific numbers of reported CE cases, hospitalizations as well as deaths due to CE in Germany in 2017 were retrieved from the official statistics [2, 36, 37]. The number of moderate CE cases was calculated by subtracting the number of hospitalized (i.e. severe) cases from the reported case numbers.
- It was assumed that the probability to develop sequelae derived from the AOK insurants would be equally applicable to all CE cases (base case model), while in a separate sensitivity analysis, probabilities from published meta-analyses [38, 39] were used.
- For GBS, age- and gender-specific parameters were incorporated into the model: The distribution of cases among the patient groups was based on published GBS incidence rates in Germany [40]. Group-specific mortality rates were calculated by dividing the reported deaths due to GBS in 2017 [37] by the total GBS cases estimated for Germany [40, 41].
- In order to extrapolate the total amount of expenses for CE in Germany, the number of reported moderate and severe CE cases were valued with the age- and gender-specific costs obtained from the claims data. An adjustment was included for multiple CE infections per patient over 12 months to approximate the costs per case.
- Similarly, each case of sequela was valued with the obtained partial costs over the assumed disease duration. As the 36-month observation period was too short to analyze the duration of long-lasting sequelae like GBS, IBD and IBS, published reports on the duration of sequelae were reviewed [42–50]. For chronic GBS and IBD, the remaining age- and gender-specific life expectancy in Germany [51] was used as the disease duration.

### Statistical analysis

The CE-specific health care utilizations and costs were analyzed in the first year of the individual study period; for sequelae, the average utilizations and costs over a 12-month period were determined considering the individual time between the first and the last diagnosis of the sequelae within the study period.

Health care cost data often show a right-skewed distribution due to a few outliers with extreme values, many zero costs and no costs less than zero. Therefore, non-parametric 95% confidence intervals of the mean costs were estimated [52–54] based on 10,000 bootstrap samples by applying the bias corrected and accelerated technique [55] using the R package boot [56]. Statistically significant associations between CE and PPI use, the presence of comorbidities as well as the development of sequelae were analyzed using the Chi-square test. Additionally, to assess whether PPI could increase the susceptibility to enteric diseases such as CE, odds ratios adjusted for age group, gender and the number of comorbidities according to the Elixhauser comorbidity index were calculated in a multivariable logistic regression analysis. Differences in the number of comorbidities and chronic conditions between patients and controls were analyzed using the Wilcoxon rank-sum test. For CE patients, existing differences or associations of health care utilizations and costs depending on disease severity, age group and gender were examined using the Wilcoxon rank-sum, Kruskal-Wallis (followed by pairwise post-hoc Dunn’s test) or Chi-square test. Normal distribution was checked using the Shapiro-Wilk test (for sample sizes ≤ 2,000) or the Kolmogorov-Smirnov-Lilliefors test (for sample sizes > 2,000). Data were analyzed in JMP 16.2.0 and R 4.1.2.

For the extrapolation of total costs in Germany in 2017 a Monte Carlo simulation was performed, in which parameters varied simultaneously depending on a priori defined distribution functions (as given in Table H in S1 File). Ten thousand iterations were completed using the R package mc2d [57]. Future costs associated with persistent sequelae in subsequent years were discounted to reflect 2017 prices with a rate of 3% (0 and 5% in the sensitivity analysis) [18].

## Results

### Study population

**Fig 2:**
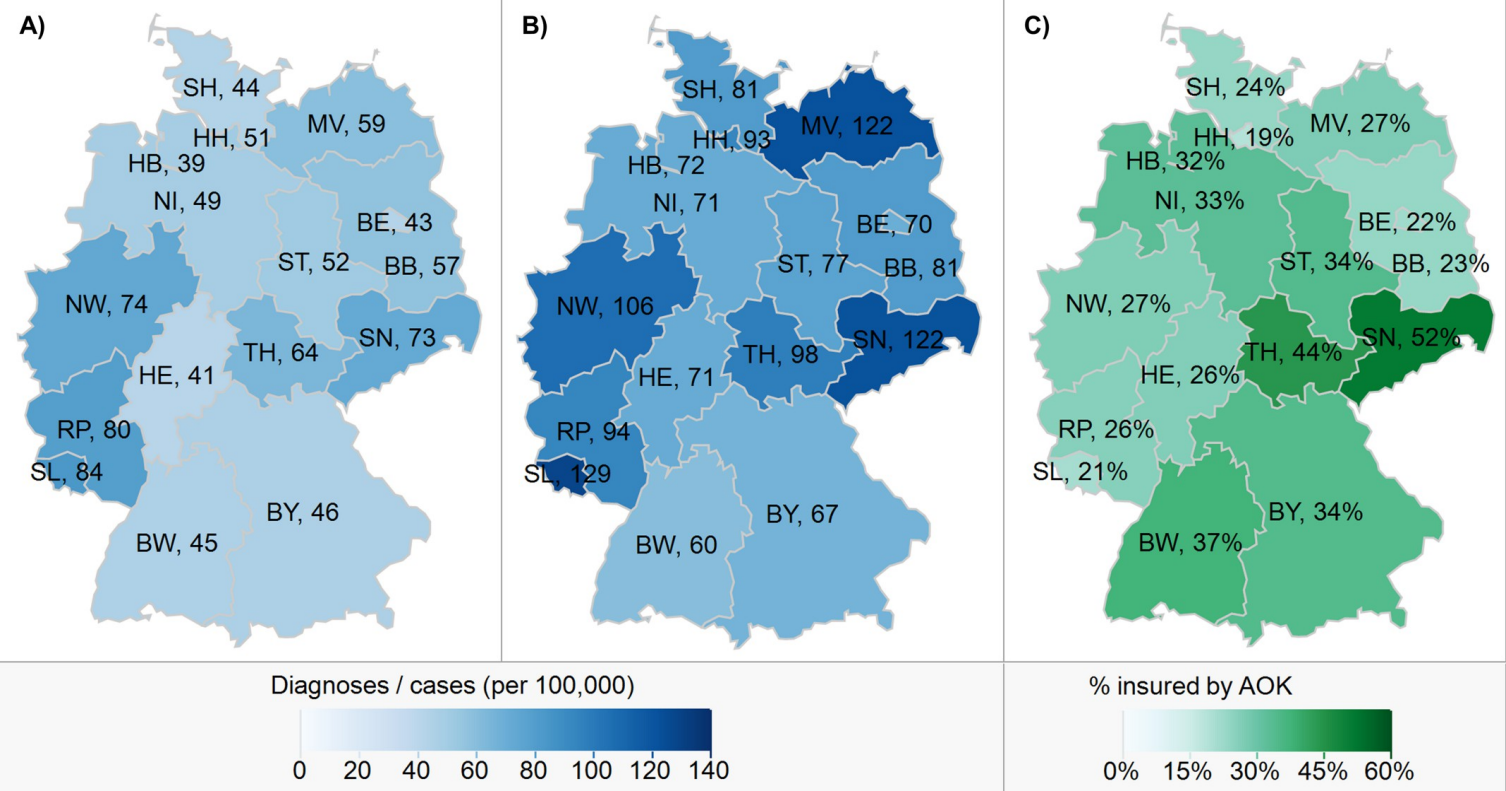
Comparison of *Campylobacter* enteritis rates between the Federal States of Germany in 2017. A) *Campylobacter* enteritis diagnoses per 100,000 AOK insurants in 2017. B) Officially reported *Campylobacter* enteritis cases per 100,000 inhabitants in Germany in 2017 according to national surveillance data [2]. C) Proportion of the population insured by AOK in 2017 according to the official statistics [22, 41]. Maps were created in JMP 16.2.0. BW: Baden-Württemberg, BY: Bavaria, BE: Berlin, BB: Brandenburg, HB: Bremen, HH: Hamburg, HE: Hesse, MV: Mecklenburg-Western Pomerania, NI: Lower Saxony, NW: North Rhine-Westphalia, RP: Rhineland-Palatinate, SL: Saarland, SN: Saxony, ST: Saxony-Anhalt, SH: Schleswig-Holstein, TH: Thuringia.

A total of 13,150 insurants had at least one confirmed or suspected outpatient or hospital-made CE diagnosis in 2017, resulting in a total of 14,453 separate new diagnoses. With 26.0 million members in mid-2017 [22], a rate of 56 CE diagnoses per 100,000 AOK insurants could be calculated. The rates were higher for male compared to female insurants in the age groups <20 and ≥60 years, whereas females showed a higher rate in the age groups 20 to 44 years (Fig A in S1 File). Gender differences were most pronounced in the age group 10 to 14 years with a 42% higher rate among male compared to female insurants (46 vs. 32 CE diagnoses per 100,000). The highest rates were found for female insurants aged 20 to 24 years and male insurants <5 years (82 and 74 diagnoses per 100,000 insurants, respectively) and the lowest rates for female insurants aged 5 to 14 years (32 per 100,000). The rates in the sixteen German Federal States ranged between 39 and up to 84 CE diagnoses per 100,000 insurants (Fig 2).

The rates of CE diagnoses of AOK insurants were consistently lower than the officially reported incidence rates for Germany [2], but showed a similar regional distribution pattern. According to both the surveillance and the claims data, rates were lowest in the 5 to 14 age group and highest for females aged 20 to 24. The largest discrepancies, with diagnoses rates less than half the officially reported incidence rates, were observed in the case of male insurants aged 30 to 44 and in Mecklenburg-Western Pomerania. The lower rates of middle-aged men resulted in rather small differences between male and female insurants aged 35 to 59, which is in contrast to the officially reported incidence rates of men showing significantly higher values than those of women from age 35 onwards.

In a sensitivity analysis, only confirmed CE diagnoses in 2017 were considered. This resulted in 12,321 separate diagnoses and a rate of 47 diagnoses per 100,000 AOK insurants.

In 43.4% of the patients, the CE diagnosis was made in hospitals. In 8.7% of the patients, the (outpatient) diagnoses were recorded as *suspected* only; consequently, these patients were excluded from further analysis. Patients who were not continuously insured throughout the study period were also excluded. Thus, overall 9,945 (75.6%) patients were available for the analysis of health care utilizations and costs.

About half of the study population was female; the median age was 47 years (Table 1). The study period for each patient and control ranged between 21 (hospital discharge in the second quarter of 2018) and 36 months (CE diagnosis in the first quarter of 2017). Comorbidities and chronic conditions were more frequent for patients than for controls (Tables A and B in S1 File); the relative discrepancy was highest for chronic IBD, with 30 times as many patients taking the disease-associated medication according to the pharmacy-based metric.

**Table 1:** Baseline characteristics (in 2017).

	Total	Study population			Subgroup with matching comorbidities		
	Patients *(n = 13*,*150)*	Patients *(n = 9*,*945)*	Controls^a^ *(n = 29*,*835)*	p value	Patients *(n = 4*,*036)*	Controls^b^ *(n = 9*,*350)*	p value
**Age** *[md (IQR)*, *min*, *max]*	45 (23–64), 0, 98	47 (24–64), 0, 97	47 (24–64), 0, 97		25 (13–38), 0, 94	23 (12–34), 0, 94	
**Age group** *[%]*							
<5 years	6.5	6.5	6.5		13.6	14.4	
5 to 14 years	6.2	6.5	6.5		13.0	13.9	
15 to 29 years	22.1	19.9	19.9		35.2	39.0	
30 to 44 years	15.1	15.0	15.0		19.3	18.4	
45 to 64 years	25.5	27.8	27.8		16.4	13.1	
≥65 years	24.8	24.3	24.3		2.5	1.3	
**Female** *[%]*	50.2	50.6	50.6		46.7	45.5	
**Continuously insured** *[%]*	82.8	100.0	100.0		100.0	100.0	
**Number of comorbidities (Elixhauser index)**^c^ *[md (IQR)*, *min*, *max]*		2 (1–5), 0, 19	1 (0–3), 0, 17	<0.001	1 (0–1), 0, 11	0 (0–1), 0, 9	<0.001
**Number of chronic conditions (pharmacy-based metric)**^d^ *[md (IQR)*, *min*, *max]*		2 (1–4), 0, 14	1 (0–3), 0, 16	<0.001	0 (0–1), 0, 8	0 (0–1), 0, 8	<0.001
**CE severity** *[%]*							
moderate	66.3	63.7			68.5		
severe	33.7	36.3			31.5		
**Use of proton pump inhibitors**^e^ *[%]*		11.2	3.3	<0.001	3.8	0.6	<0.001
**Previous diagnoses**^f^ of *[%]*							
CE		7.78	0.00		5.50	0.00	
REA		0.01	0.00	0.083	0.02	0.00	0.128
GBS		0.06	0.00	<0.001	0.02	0.00	0.128
IBD: CD		1.18	0.00	<0.001	0.62	0.00	<0.001
IBD: UC		1.15	0.00	<0.001	0.52	0.00	<0.001
IBS		2.64	0.00	<0.001	1.66	0.00	<0.001
**New diagnoses**^g^ of *[%*, *md (min-max) months after CE]*							
REA		0.12 %, 0 (0–3)	0.00	<0.001	0.02	0.00	0.128
GBS		0.06 %, 1 (0–21)	0.00	<0.001	0.12	0.00	<0.001
IBD: CD		1.44 %, 0 (0–28)	0.01	<0.001	1.36	0.00	<0.001
IBD: UC		1.86 %, 0 (0–30)	0.00	<0.001	1.66	0.00	<0.001
IBS		3.39 %, 6 (0–33)	0.01	<0.001	2.53	0.01	<0.001

The Chi-square and Wilcoxon rank-sum test were used to analyze statistically significant associations with CE and differences between patients and controls, respectively. CD: Crohn’s disease, CE: *Campylobacter* enteritis, GBS: Guillain-Barré syndrome, IBD: inflammatory bowel disease, IBS: irritable bowel syndrome, IQR: interquartile range, md: median, REA: reactive arthritis, UC: ulcerative colitis.

^a^ Three controls per patient, matched by age, gender and state of residence.

^b^ One to three controls per patient, matched by age, gender, state of residence and existing comorbidities (in terms of either the Elixhauser comorbidity index [27, 28] or the pharmacy-based metric with the ATC algorithm [29], without *pain*).

^c^ Comorbidities during the first year of the individual study period were assessed by applying the Elixhauser comorbidity index [27, 28]. The full list of existing comorbidities is displayed in Table A in S1 File.

^d^ Chronic conditions during the first year of the individual study period were assessed by applying the pharmacy-based metric with the ATC algorithm [29]. The full list of existing chronic conditions is displayed in Table B in S1 File.

^e^ Prescriptions of proton pump inhibitors within four weeks prior to the individual study period according to the pharmacy dispensing date.

^f^ Diagnoses made within 12 months prior to the individual study period.

^g^ Diagnoses made during the individual study period were considered as new, provided that no previous diagnoses were reported 12 months prior to the study period; for patients, the median time between the first CE diagnosis and the first diagnosis of sequelae is shown.

The use of PPI was positively associated with a CE diagnosis: The adjusted odds of a pharmacy dispensing of prescribed PPI within the last four weeks was 3.2 times (95% CI 2.9–3.5) higher in patients than in controls (Table C in S1 File).

Based on the CE-specific health care utilization, 63.7% of the patients were categorized as moderate and 36.3% as severe cases. More than one CE diagnosis during the study period was made for 8.8% of moderately and 1.3% of severely affected patients; the median number of diagnoses was one with a maximum of eleven diagnoses within 36 months. A second CE diagnosis was recorded on average six months (IQR: 4–9, min-max: 3–34) after the first diagnosis. In the first year of the study period, there was an average of 1.11 and 1.01 CE diagnoses per patient with moderate and severe CE, respectively.

Previous CE diagnoses within 12 months prior to the study period were reported for 11.8 and 0.7% of the patients with moderate and severe CE, respectively. In general, the prevalence of previous or persistent REA, GBS, IBD and IBS was low (Table 1). Moreover, 6.3% of the CE cases developed a new sequela concurrent with or after their CE, ranging between 0.06% (GBS) and 3.4% (IBS) for each sequela. REA and IBD occurred more often in patients with severe than in patients with moderate CE (REA: 0.22 vs. 0.06%, one-tailed Fisher’s exact test: p = 0.032; CD: 1.9 vs. 1.2%, p = 0.001; UC: 2.5 vs. 1.5%, p < 0.001). While REA was diagnosed within three months after the CE diagnoses in all affected patients, IBS diagnoses were made on average after six months (Table 1). The time between the first and the last diagnosis amounted to a median of one month for REA (range: 0 to 20 months), two months for IBD and IBS (range: 0 to 35 months) and ten months for GBS (range: 0 to 31 months). In 0.54% of the patients, more than one sequela was diagnosed: 0.31% developed both CD and UC, followed by diagnoses of either UC or CD along with IBS (0.17 and 0.11%, respectively). Diagnoses of GBS, UC and IBS as well as REA with IBS were recorded in one patient each.

### Health care utilizations and work incapacities

#### Campylobacter enteritis

Health care utilizations associated with CE diagnoses differed significantly depending on disease severity (Table 2). The percentage of outpatient consultations with GP or specialists and of patients taking antibiotics was higher for moderate CE, whereas patients with severe CE received more frequently prescriptions of analgesics and spasmolytics and were more frequently hospitalized (by definition). Both moderate and severe CE patients had most frequently outpatient consultations with GP and second most frequently with internists, whereas patients <15 years mostly consulted pediatricians. Further physicians involved in outpatient medical care were mainly laboratory specialists (claims of 1.3 and 0.3% of moderate and severe CE patients, respectively). Taken together, 4,458 patients had a CE-associated hospital stay, 19.0% of them being linked with a secondary CE diagnosis (i.e. moderate CE). Hospital stays with principal CE diagnoses lasted on average five days, while female and older patients experienced a longer duration. A second CE-associated hospital stay was claimed in 32 cases, and for one patient a third hospital stay was recorded. Readmission of severely affected patients occurred after a median of two months (range: 0 to 26 months) after their first hospital stay.

**Table 2:** Health care utilizations and work incapacities associated with diagnoses of *Campylobacter* enteritis or sequelae over a 12-month period.

	*Campylobacter* enteritis (CE)			Sequelae^a^				
	Moderate CE (n = 6,336)	Severe CE (n = 3,609)	p value	Reactive arthritis (n = 12)	Guillain-Barré syndrome (n = 6)	Crohn’s disease (n = 143)	Ulcerative colitis (n = 185)	Irritable bowel syndrome (n = 337)
**Outpatient medical care** *[%]*								
GP	51.7	11.9	<0.001	16.7	16.7	44.1	54.1	48.4
pediatrician	10.5	1.2	<0.001	0.0	0.0	6.3	5.4	1.8
internist	25.1	4.7	<0.001	8.3	66.7	38.5	30.8	43.3
other physicians	3.9	0.6	<0.001	33.3	16.7	16.1	17.3	13.4
provided in hospitals	0.2	0.3	0.202	0.0	16.7	6.3	4.3	2.4
**Inpatient medical care**^b^ *[%]*	0.0	100.0	<0.001	25.0	66.7	7.7	13.0	2.1
*stays [md (min-max)]*		1.0 (1.0–2.0)		1.0 (0.6–1.0)	0.7 (0.4–1.0)	0.9 (0.4–2.0)	1.0 (0.4–3.0)	1.0 (0.5–1.0)
*days per stay [md (IQR*, *min-max)]*		5.0 (4.0–7.0, 1.0–53.0)		6.0 (4.0–22.0, 4.0–22.0)	21.0 (14.3–104.3, 13.0–131.0)	7.5 (5.3–15.8, 2.0–20.0)	8.0 (4.0–13.0, 2.0–90.0)	5.0 (3.0–5.0, 2.0–7.0)
**Rehabilitation** *[%]*	0.0	0.0		0.0	33.3	0.0	0.5	0.0
*stays [md (min-max)]*					0.4 (0.4–0.4)		0.5	
*days per stay [md (min-max)]*					111.5 (61.0–162.0)		20.0	
**Prescribed medication**^c^ *[%]*								
antibiotics	13.1	8.7	<0.001					
analgesics/spasmolytics	18.7	24.2	<0.001					
oral rehydration solution	1.7	1.3	0.119					
**Incapacities for work** *[%*^*d*^*]*	18.9	57.5	<0.001	25.0	50.0	30.2	31.1	16.4
*days [md (IQR*, *min-max)]*	8.0 (4.0–14.0, 1.0–247.0)	3.0 (1.0–7.0, 1.0–207.0)	<0.001	3.0 (1.0–5.0, 1.0–5.0)	128.7 (46.0–211.4, 46.0–211.4)	5.0 (3.0–12.0, 0.6–162.0)	5.5 (2.8–13.8, 0.5–144.5)	2.0 (1.0–6.8, 0.6–38.0)

The percentage of patients utilizing the respective service or of patients who were incapacitated for work (%) and median (IQR, min-max) number of days or stays of utilizing patients are given. Existing differences or associations depending on CE severity were examined using the Wilcoxon rank-sum or Chi-square test, respectively. IQR: interquartile range, md: median.

^a^ Average health care utilizations associated with sequelae per 12 months were determined considering the individual time between the first and the last diagnosis of the respective sequela.

^b^ Only hospitalizations with principal diagnoses were considered to be disease-associated and are displayed.

^c^ Considered to be CE-associated according to national medical treatment guidelines, as prescriptions of medication are not directly diagnosis-linked in the claims data.

^d^ Proportion of patients aged 15–64 years; days refer to patients incapacitated for work according to incapacity certificates; short-term absences might not have been recorded in the claims data.

For moderate CE patients aged 15 to 64 years, work incapacities lasted an average of eight days, which was longer than the three days for hospitalized patients. However, the proportion of patients with work incapacities was higher in the case of patients with severe CE than in the case of patients with moderate CE (57.5 vs. 18.9% of patients aged 15 to 64 years).

CE-associated health care utilizations and incapacities for work differed significantly between age group and gender. A comprehensive listing of age- and gender-specific utilizations is provided in Table D in S1 File.

#### Sequelae

About half of the IBD and IBS patients had sought outpatient GP consultations; in the case of REA and GBS patients, the proportions were lower (Table 2). Internist consultations were also frequent for patients with sequelae other than REA. Concerning REA and GBS patients, neurologists and orthopedists were among the most frequently consulted other physicians (8% each of REA and 17% of GBS patients), while gynecologists were particularly involved in the outpatient medical care of IBD and IBS patients (3–4% of patients). The highest proportion of hospitalizations was among GBS patients, with an average stay of 21 days every one and a half years. Inpatient rehabilitations were seldom and were only seen in two GBS cases and one UC patient with one stay of 112 and 20 days, respectively, within two years. Work incapacities were most common and longest observed in the case of GBS patients, while patients with other sequelae had rather short absences from work of up to five days per 12 months (median).

### Cost of illness

#### *Campylobacter* enteritis

The estimation of CE-specific costs of outpatient medical care and prescribed medication was based on 4,036 patients with at least one control matched by age, gender, state of residence and existing comorbidities (total of 9,350 controls, i.e. on average 2.3 controls per patient). Insurants included in the regression analyses were younger and had fewer comorbidities and chronic conditions than the total study population (Table 1). The regression outputs are shown in detail in Tables E and F in S1 File. The estimated per patient mean disease-specific costs of outpatient medical care over a 12-month period were higher for patients with moderate CE than for patients with severe CE, whereas costs for inpatient medical care, prescribed medication and indirect costs were higher for severe CE (Table 3). Costs differed considerably between age groups: Patients ≥65 years developed the highest direct costs, whereas indirect costs were primarily incurred by patients aged 15 to 64 years (Table G in S1 File). There were also statistically significant gender differences, with women showing slightly higher costs of outpatient medical care and prescribed medication, while men with severe CE tended to have higher indirect costs.

**Table 3:** Mean disease-related costs per patient over a 12-month period [in € (95% confidence intervals)].

	*Campylobacter* enteritis (CE)			Sequelae^a^				
	Moderate CE	Severe CE	p value	Reactive arthritis	Guillain-Barré syndrome	Crohn’s disease	Ulcerative colitis	Irritable bowel syndrome
**Direct costs**	208 (207–210)	2,355 (2,315–2,405)	<0.001	748 (244–1,727)	11,161 (4,718–20,683)	251 (121–477)	462 (281–1,053)	41 (20–82)
outpatient medical care^b^	177 (176–178)	95 (94–96)	<0.001					
outpatient medical care provided in hospitals	0 (0–0)	0 (0–0)	0.270	0	49 (0–97)	19 (4–76)	9 (3–27)	6 (3–14)
inpatient medical care	0	2,227 (2,187–2,278)	<0.001	748 (244–1,727)	8,620 (2,976–19,096)	231 (112–446)	453 (271–999)	35 (14–73)
rehabilitation	0	0		0	2,493 (0–4,986)	0	0	0
prescribed medication^b^	31 (31–32)	33 (32–33)	<0.001					
**Indirect costs** ^**c**^	316 (286–352)	475 (429–532)	<0.001	135 (0–472)	11,560 (0–32,612)	978 (475–2,263)	731 (455–1,363)	180 (111–309)
**Total**	**524 (495–560)**	**2,830 (2,769–2,905)**	<0.001	**883 (279–1,786)**	**22,721 (7,461–52,403)**	**1,229 (673–2,679)**	**1,192 (785–2,422)**	**221 (147–350)**

Costs of outpatient medical care provided in hospitals, inpatient medical care and rehabilitations associated with diagnoses of *Campylobacter* enteritis or sequelae; costs of outpatient medical care and medication estimated as additional costs in comparison to controls (see footnote b); indirect costs correspond to work incapacities monetized with the average labor costs. Costs are given as mean and 95% confidence intervals (based on 10,000 bootstrap samples). Existing differences between patients with moderate and with severe CE were examined using the Wilcoxon rank-sum test.

^a^ In case of diseases lasting >12 months, the average costs per 12 months were determined considering the individual time between the first and the last diagnosis of the respective sequela.

^b^ The regression outputs are shown in Tables E and F in S1 File. The observed total median costs of outpatient medical care were € 262 for patients with moderate CE, € 184 for patients with severe CE and € 99 for controls. The observed total median costs of prescribed medication were € 46 for patients with moderate CE, € 43 for patients with severe CE and € 21 for controls. For sequelae, estimation of disease-specific costs of outpatient medical care and of prescribed medication was not performed due to small case numbers.

^c^ Indirect costs of patients aged 15–64 years per 12 months: moderate CE: € 473 (427–525); severe CE: € 850 (775- 956); reactive arthritis: € 202 (0–674); Guillain-Barré syndrome: € 17,339 (0–42,720); Crohn’s disease: € 1,206 (608–2,709); ulcerative colitis: € 913 (570–1,713); irritable bowel syndrome: € 226 (142–385).

**Fig 3:**
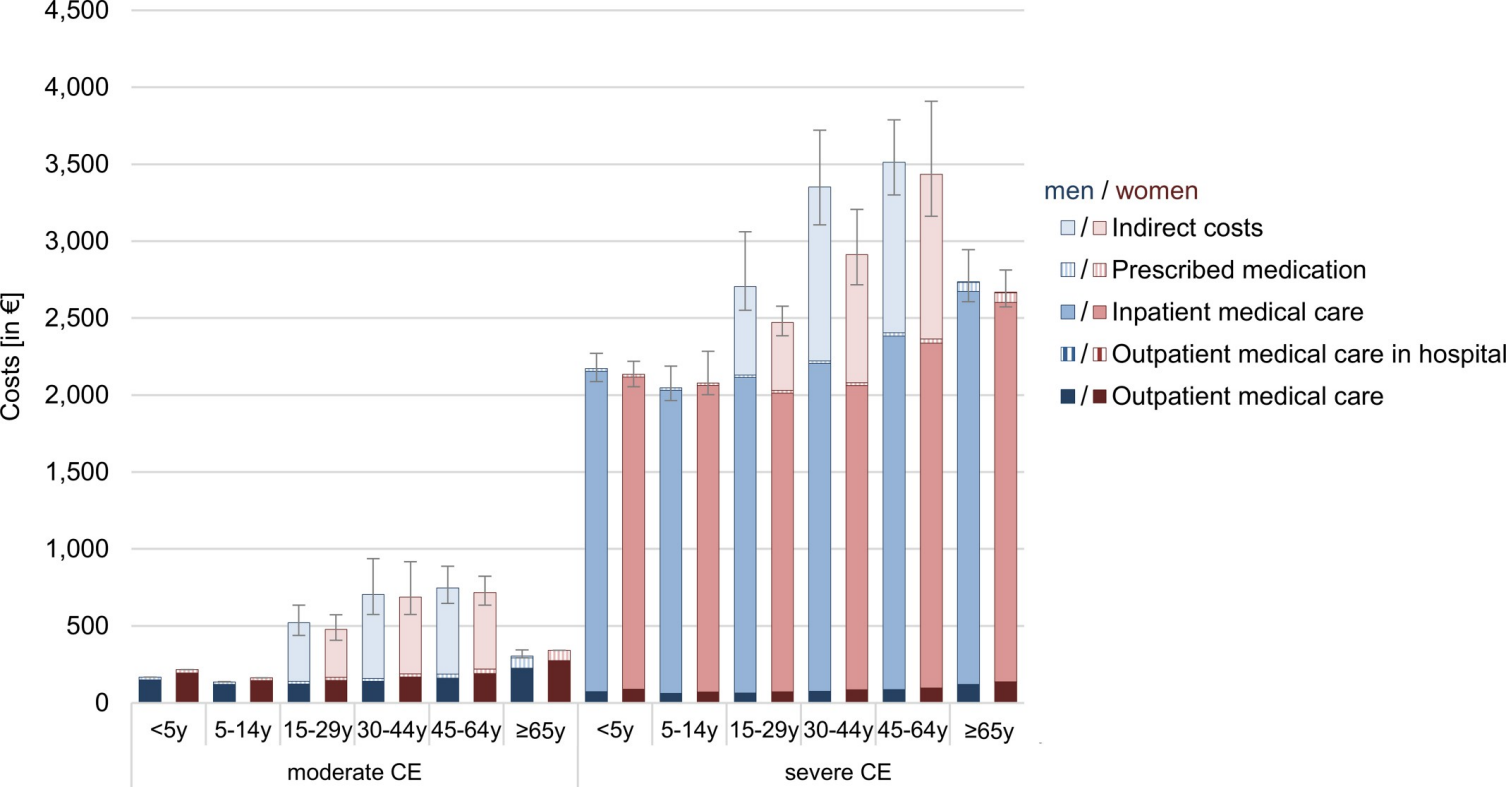
Mean costs of *Campylobacter* enteritis by age group and gender over a 12-month period. Costs per patient in €. Error bars indicate 95% confidence intervals of total costs. CE: *Campylobacter* enteritis, y: years.

Total costs of CE summed up to € 524 (95% CI € 495–560) per patient with moderate CE and € 2,830 (€ 2,769–2,905) per patient with severe CE over a 12 month-period. Total cost of illness was lowest for patients between five and 14 years of age and highest for the age group 30 to 64 for both moderate and severe CE (Fig 3). Direct medical costs accounted for 40% of total costs for moderate CE, while for severe CE the proportion was 83%, due to the high costs for inpatient medical care. Hospitalizations constituted the largest cost factor for severely affected patients, accounting for 79% of the total costs. In contrast, regarding patients with moderate CE, productivity losses accounted for the largest share of 60%.

#### Sequelae

The costs of hospital medical care and rehabilitations as well as the indirect costs associated with a diagnosis of GBS summed up to € 22,721 (mean, 95% CI € 7,461–52,403) per patient over a 12-month period. Mean disease-related costs for REA, CD, UC and IBS were considerably lower (between € 221 for IBS patients and € 1,229 for CD patients per 12 months, Table 3).

### Extrapolation of total costs in Germany

**Fig 4:**
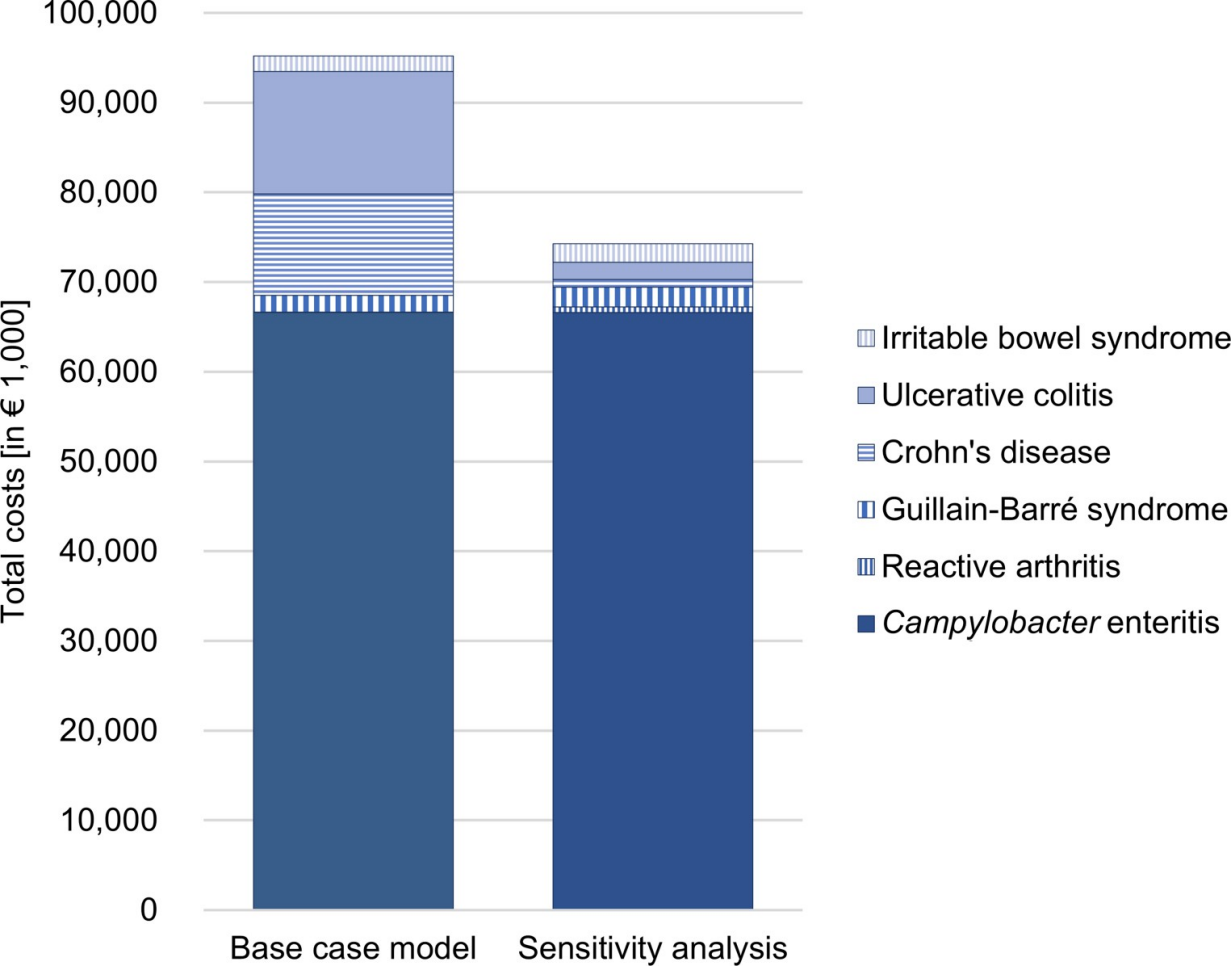
Extrapolated total costs associated with *Campylobacter* enteritis cases in 2017 including sequelae in Germany. Extrapolation is based on *Campylobacter* enteritis cases, hospitalizations and deaths as officially reported for Germany in 2017 [2, 36, 37]. In the sensitivity analysis, the frequencies of sequelae according to published meta-analyses [38, 39] were used. Total costs were estimated by Monte Carlo simulation with 10,000 iterations.

The age- and gender-specific mean costs per patient per 12 months obtained through the claims data were used to extrapolate the total costs to all CE cases in Germany. In 2017, there were 69,476 officially reported CE cases according to national surveillance data [2]. Official statistics revealed 13,159 hospitalized cases with principal CE diagnoses and eight deaths due to CE in 2017 [36, 37]. The extrapolated mean costs were € 95.19 million (95% CI € 95.10–95.28, Fig 4). Sequelae had a high impact on the total costs; they accounted for 30.0% of total costs. Chronic IBD in particular contributed to the high costs of sequelae. The extrapolation of future costs of CE due to sequelae in subsequent years was carried out with different discounting rates, which resulted in considerable differences in the total costs (minus € 12.09 million if discounted at 5% and plus € 52.81 million if undiscounted, Table I in S1 File).

In the sensitivity analysis, the frequency of sequelae was based on published meta-analyses. The largest relative difference in the extrapolated number of CE cases developing sequelae was found for REA with 1,193 cases compared to 85 in the base case model, whereas for GBS the estimated case numbers were similarly low (Table I in S1 File). Especially the reduced probabilities for IBD led to a significant decrease in the mean estimate of total costs of € 74.25 million (95% CI € 74.20–74.31).

## Discussion

This is the first comprehensive analysis of the health care utilizations and costs of a large group of CE patients in Germany. The analyzed claims data represent more than one third of the statutorily insured persons and 31.4% of the population in Germany [22, 41]. Nevertheless, the calculated **rate of CE diagnoses** among insurants was lower than the 84 cases per 100,000 inhabitants officially reported in Germany in 2017 according to national surveillance data [2, 3]. If a shorter interim period of 30 instead of 90 days between separate CE diagnoses would be used or if all CE diagnoses would be counted as new diagnoses, the rate would still remain below the official incidence rate (59 and 67 CE diagnoses per 100,000 insurants, respectively). However, the latter assumptions might very likely lead to double counting, as some hospitalized patients may have received outpatient follow-up treatments, and some outpatient diagnoses were subsequently positive over several quarters, even though CE is considered an acute infection, which usually lasts only a few days to weeks [4].

Interestingly, AOK insurants appeared to be more frequently hospitalized: While 25.6% of the claimed CE diagnoses in 2017 were principal diagnoses made in hospitals, official statistics revealed 13,159 hospitalizations due to principal CE diagnoses in Germany corresponding to 18.9% of the officially reported 69,476 CE cases in 2017 [2, 36]. Therefore, we suppose that outpatient CE diagnoses in particular are missing in the claims data. The reason might be that some physicians initially diagnosed general gastroenteritis or suspected CE, but did not further specify or correct it as a confirmed *Campylobacter* enteritis (code A04.5) later on, even if positive laboratory results were made available. By taking only confirmed CE diagnoses into account, the rate of CE diagnoses was considerably lower (47 per 100,000 AOK insurants). Moreover, in some cases, it seems possible that medical services were not included in the claims data, as they were not relevant for accounting or were provided outside the scope of the SHI-accredited care.

We investigated whether the AOK insurants can be considered representative for Germany–both in general and in terms of CE cases: No major differences in the distribution of age groups and gender between the 26 million AOK insurants and the general population of 83 million were found, although the proportion of inhabitants insured by AOK varied across the Federal States (as illustrated in Fig 2C) [22, 41]. Regarding the CE cases, AOK insurants showed a similar distribution in terms of age, gender and region as the officially reported cases according to national surveillance data. Therefore, we assume that the claims data give a relatively accurate picture of campylobacteriosis in Germany. The regional differences within Germany might be due to specific regional risk factors (e.g. higher exposition to farm animals in rural regions), the demographic structure [58] and the associated health-seeking behavior. Reasons for the higher incidence in female compared to male young adults include human-to-human transmissions from infants and the higher probability of preparing and consuming poultry meat; the higher incidence rates in children <5 years compared to older children are most likely due to the not yet fully developed immune system, specific risk factors such as animal contact and inadequate hand hygiene [58] and presumably also a higher probability of consulting a physician in case of CE-specific symptoms.

The analysis of **health care utilizations** revealed higher proportions of moderate than severe CE cases using outpatient medical care or medication due to their CE. Unfortunately, it was not possible to reliably analyze the number of outpatient visits associated with CE diagnoses based on the claims data, as outpatient diagnoses are only reported quarterly and are not linked to single outpatient medical services. Patients with severe CE saw a physician predominantly during their hospitalizations. Similarly, medication administered during hospital stays is covered by the German diagnosis-related groups system for hospital financing and does not appear as separate codes within the claims data. Therefore, the proportion of severe CE patients taking antibiotics, analgesics or spasmolytics or receiving oral rehydration solutions might be underestimated. Even for moderate CE cases, the data may not fully reflect the actual proportion of patients taking medication: We assumed that any prescribed medication was CE-related, if this treatment was i) recommended in medical treatment guidelines and ii) occurred within the same annual quarter. However, this might not necessarily be the case. Moreover, there might be discrepancies between the recommended and the actual treatment of CE. For example, erythromycin was still a frequently taken antibiotic in a survey of CE cases in Germany between 2011 and 2014 [4], but medical treatment guidelines explicitly advised against its prescription shortly thereafter due to side effects [30]. A separate analysis regarding erythromycin use showed that still 4.0% of the AOK insurants were taking this antibiotic during the quarter of their CE diagnoses. However, these cases are not included in Table 2.

The analysis also showed that the median duration of work incapacities of severe CE patients was shorter than their hospital stays, which seems like a self-contradictory result. This underestimation can be explained by i) certificates of incapacity for work are usually issued to employees only (and not to non-working patients, such as students, unemployed or retired individuals), ii) it is possible that not all employees submitted a separate copy of the certificate to the health insurance as required, and iii) depending on the employer, no certificate is needed until the fourth day of sick leave. Short-term absences that were not recorded within the claims data are particularly likely in the case of not severely affected patients. Therefore, the duration of work incapacities in patients with moderate CE might be overestimated due to the missing short-term absences, which also explains their longer median duration compared to patients with severe CE. The true proportion–and in the case of severe CE also the duration–of sick leaves are most probably higher than the numbers extracted solely from the claims data. This assumption is supported by a recent survey of 1,800 CE cases in Germany, in which a significantly higher percentage of self-reported work absences of 79%, including those of working parents with sick children, was observed [4].

Beside the absences from work, presenteeism–i.e. going to work despite reduced productivity for example due to a disease–is known to increase the indirect costs. Presenteeism is rarely included in cost of illness studies until now, also because there is no consensus on how it can be measured appropriately [59]. The same is true for our study, as claims data do not provide information on presenteeism. The productivity losses due to long-lasting and chronic sequelae such as IBS [60] are therefore probably underestimated, whereas presenteeism might be of minor importance in acute and short-term CE.

Estimates of the **direct and indirect costs of *Campylobacter* enteritis** in other countries range from a total of € 350 per CE case (not differentiating between moderate and severe) in the Netherlands to € 540 in Sweden to $ 1,020 in the USA [12, 13, 15]. The cost categories taken into account in these studies were similar to our analysis, with the exception of medication costs, which were excluded in one study [12], and over-the-counter medication, travel costs and/or informal care, which were additionally included in the other two studies [13, 15]. In the present study, the COI of CE patients was analyzed over a 12-month period. Accordingly, the estimates do not reflect costs per CE case but costs per patient per 12 months. Nevertheless, costs per CE case are similar to the costs presented, as moderately and severely affected patients had on average 1.11 and 1.01 diagnoses in 12 months, respectively.

Severe CE caused 4.4 times higher costs than moderate CE. In contrast, the modeling approach for the USA suggests 23 times higher costs in severe compared to moderate CE cases ($ 620 vs. $ 14,850) with a similar definition of disease severity [12]. These differences can be explained by both country-specific conditions and differences in the methodology. Most of the COI studies so far assume a certain CE-associated health care utilization based on population surveys, valued with national prices. One exception is a study from Switzerland, in which invoice data of outpatient medical care of 41 CE patients was analyzed to validate one part of their modeling approach [14]. The present analysis of claims data of almost 10,000 CE patients provides a comprehensive view of the actual health care utilizations (although limited to benefits relevant for accounting), real payments by both health insurance and patients, and sick leaves according to submitted certificates of incapacity for work in Germany.

However, some assumptions had to be made in order to evaluate disease-specific utilizations and costs, e.g. the CE-attribution of benefits that are not diagnosis-linked in the claims data. To prevent an overestimation of hospital stays due to other–probably more care- and cost-intensive–diseases, hospital stays associated with secondary CE diagnoses were not considered disease-related and consequently not analyzed.

Although one would expect that detection of the pathogen or respective antibodies or antigens following a physician’s consultation is a prerequisite for making *confirmed* diagnoses, we could not identify outpatient consultations nor laboratory tests for some of the moderate CE patients. Possible explanations for this observation could be that i) not all outpatient medical services were claimed separately due to lump sum compensations, and ii) some codes related to single benefits were not identifiable, as different additional regional contracts exist between the association of SHI physicians and the health insurance, and/or due to potential entry errors.

A major limitation of analyzing the COI according to German claims data is the missing link between the underlying diagnoses and the costs of outpatient medical care and of prescribed medication. The CE-specific costs were therefore estimated in a subgroup of patients and controls with matching comorbidities. The additional risk adjustment was necessary, as the randomly selected controls seemed to be healthier according to the Elixhauser comorbidity index and the pharmacy-based metric. The difference in comorbidities might also be induced by the health-seeking behavior of patients: Insurants with comorbidities are more likely to consult a physician due to other health problems, which also increases the probability of getting tested for *Campylobacter*. Although the subgroup was younger than the total study population, the excess costs could be estimated for all six age groups. Unfortunately, a comparable approach for sequelae was not possible due to small case numbers. This is also the reason why there was no subgroup analysis by age for sequelae, although symptoms and treatment of GBS, IBD and IBS differ between children and adults [45, 46, 61, 62]. Especially GBS and REA were rare sequelae, with only six and twelve diagnosed patients in our dataset, respectively. Therefore, the observed health care utilizations and costs could possibly be skewed by extreme values, which is reflected in the wide ranges and comparatively large confidence intervals of the results.

Health care costs were analyzed from a **societal perspective**. However, most costs reflect the payer’s perspective, as claims data were used. By including patients’ co-payments where available and by monetizing the incapacities for work, an approximation of the societal costs could be achieved [63]. Nevertheless, not all relevant cost factors could be taken into account due to a lack of data, e.g. costs of outpatient rehabilitation, rehabilitation of working patients (usually the responsibility of the pension insurance), therapeutic devices and remedies or productivity losses due to presenteeism, and in case of sequelae, costs of outpatient medical care and medication. In addition, hospital investment costs by the German Federal States [63] are not reflected in the estimated costs based on claims data. The same applies to direct costs paid by patients, e.g. for over-the-counter medication, for travel to health care facilities or for taking care of ill children or elder relatives. While some costs might be of minor importance, the omission of costs of outpatient medical care, medication, devices and remedies for sequelae leads to an underestimation of the real costs: IBD and IBS patients mainly sought outpatient rather than inpatient medical care according to the claims data. GBS patients often need devices and physiotherapy supporting mobility and respiration [61, 64] and severe GBS, REA and IBD cases require a costly immunomodulatory treatment [44, 65, 66].

In a recent claims data analysis from Germany, mean total costs of CD and UC per year were € 10,100 and € 8,770, respectively, and medication costs were responsible for around 50% of total COI [67]. However, only patients who had received an initial treatment with immunosuppressants, anti-tumor necrosis factor α or anti-integrin therapies were included in that analysis, whereas in the present study mild IBD cases were considered as well. Therefore, the health care utilizations and work incapacities of CD and UC patients in the present study were considerably lower than those analyzed by Wilke et al. (2020), who described that >90% of the patients had a GP visit, >40% were hospitalized and >50% were absent from work due to IBD [67]. For IBS, German claims data revealed costs of outpatient and inpatient medical care as well as prescribed medication of newly diagnosed insurants that exceeded the direct costs in age- and gender-matched controls by € 982 in 2017 [68]. However, this cost difference was not adjusted for comorbidities, which were more frequent in IBS patients. Similar COI studies from Germany for REA or GBS are not available. The use of general COI estimates for sequelae might not be appropriate, as the disease course and prognosis might differ in cases with and without a preceding infection: Post-infectious IBS could be milder than IBS alone [69] and *Campylobacter*-associated GBS seems to be more severe and of longer duration than other GBS [43, 70]. Therefore, the obtained partial costs of sequelae were used for the extrapolation of total costs in Germany, although there were some limitations, as discussed.

The study period was limited to 36 months to generate current estimates of costs incurred in 2017/2018, but also to obtain some information about the **course of CE and the development of sequelae**. The proportion of patients who developed GBS and IBS are similar to previously published rates of 0.07 and 4.0%, respectively [38]. In contrast, the incidence of REA was considerably lower and IBD occurred significantly more often than the previously reported 1.7 to 2.9% for REA and 0.05 to 0.45% for IBD [38, 39]. With 0.08% an even lower probability of REA within 12 months after CE were recently reported for England, based on health records [71]. Diagnosis rates of REA within the health care system might be lower than in prospective (outbreak) studies, as symptoms are temporary and patients with mild symptoms might not seek health care and thus remain undiagnosed. According to the claims data, REA was diagnosed on average within one month after the CE diagnosis, which is similar to the findings of the previously mentioned English study [71].

The observed new IBD diagnoses could overestimate the actual number of CE-associated cases, as the distinction between a self-limiting gastrointestinal disease caused by *Campylobacter* and an initial manifestation of IBD may be difficult [45, 46]. Moreover, in the present study suspected (and not yet confirmed) diagnoses of sequelae were also counted as new cases. There is an ongoing discussion on the causal, biologic plausible relationship between CE and post-infectious IBS and IBD [6, 7, 69], which is why disease models of campylobacteriosis differ regarding the inclusion of IBS and IBD, e.g. [10, 11]. In the present study, we used the convenient assumption that a temporal connection between a *Campylobacter* enteritis and a sequela supports a causal relationship, but this might not always be the case.

There was a high number of CE patients with preexisting IBD (n = 170) and IBS (n = 181), which were not considered to be CE-related in our analysis. This could explain the significantly higher proportion of patients taking medication for chronic IBD according to the pharmacy-based metric compared to controls.

The duration of the respective sequelae was approximated by using the first and the last day of the disease-specific utilizations available from the claims data. However, this probably does not reflect the average disease duration of sequelae due to small case numbers, the limited study period of up to 36 months and the possibility of missing diagnosis dates in the claims data. While for REA the timespan between the first and the last diagnosis was found to be maximally 20 months, the treatment of other sequelae might have continued after the 31^st^ of December 2019. Moreover, some providers might claim health services with a delay, so that sequelae-related services from 2019 may be missing in the dataset. Therefore, published results and assumptions on the duration of sequelae were used for the **extrapolation of total COI** in Germany. The total costs associated with CE cases in 2017 varied considerably, depending on the assumed probability to develop sequelae. The observed higher frequencies of CD and UC for AOK insurants compared to published figures [38] led to significant higher total costs in the base case model, as these chronic diseases cause costs until death. In comparison, according to the results of the sensitivity analysis, the sequelae-related direct and indirect costs accounted for 10% instead of the 30% share of the total costs. In other studies, which included the costs of REA, GBS, IBS and/or IBD, the COI approximately doubled [12, 13, 15]. In case that IBD and IBS were omitted as sequelae, total costs of campylobacteriosis in Germany resulted in similar amounts of € 68.51 (base case model) and € 69.44 million (sensitivity analysis), of which less than 5% were due to the occurrence of REA and GBS.

To estimate the total societal economic burden of campylobacteriosis in 2017, we assumed that all officially reported CE cases in Germany caused the same average amount of expenses according to their assignments into the respective patient groups (defined by CE severity, age and gender). This might not completely reflect the situation, as 12.8% of the population in Germany is not statutorily insured [72] and medical services are priced differently by private insurances. Similarly, the amount of co-payments by patients might differ. The generalizability of costs estimated on the basis of the claims data of AOK insurants could also be affected by differences in the distribution of morbidities and the social structure between different SHI [19]. Moreover, only officially reported cases were considered in the extrapolation. Patients with mild symptoms not seeking health care or cases with no or no successful laboratory confirmation of *Campylobacter* were not included in the extrapolation. In Germany, the estimated number of cases might be four to nine times as high as the officially reported cases [73, 74]. Most of the underestimated cases do not incur CE-associated health care costs, because CE is supposed to be rather under-ascertained than underreported in Germany [74], but costs due to short-term sick leaves or expensive sequelae are possible. The actual economic burden of campylobacteriosis in Germany is therefore probably higher than that described here.

It is estimated that 20 to 30% of the human CE cases can be attributed to the preparation, handling and consumption of contaminated chicken [8]. Another risk factor for CE appears to be the **medication with PPI**, which explained 10% of CE cases in a German case-control study with an adjusted odds ratio of 1.9 [4]. The claims data revealed an adjusted odds ratio 1.7-times as high. In a recent meta-analysis, an even higher risk with a pooled odds ratio of 5.1 was calculated based on studies from the Netherlands and the United Kingdom [75]. In the present study, the pharmacy dispensing of prescribed PPI was used and additionally an adjustment regarding the number of comorbidities was included, while in the two other recently published studies, the actual use predominantly in the previous four weeks (according to medical records or self-reports) was evaluated [4, 75].

In this study, detailed information on the health care utilizations and associated costs of CE patients in Germany are presented for the first time. The results show that CE is associated with a substantial economic burden–even if IBD and/or IBS are not considered as sequelae of CE. As far as we know, this study is the largest claims data analysis regarding CE, with almost 10,000 cases included. It is even more noteworthy that PPI could be confirmed as a risk factor for CE on the basis of this large data set.

## Supporting information

S1 File Supplementary figures and tables. (PDF)
