# Supplementary material for: Health care utilizations and costs of Campylobacter enteritis in Germany: A claims data analysis
Source: PLoS One. 2023 Apr 5;18(4):e0283865. doi: 10.1371/journal.pone.0283865 (PMC10075411; doi:10.1371/journal.pone.0283865)
Supplement: S1 File — (PDF) [file pone.0283865.s001.pdf]

Supplementary figures and tables for

Health care utilizations and costs of *Campylobacter* enteritis in Germany: a claims data analysis

Elisabeth Schorling, Sonja Lick, Pablo Steinberg and Dagmar A. Brüggemann

Fig A. Comparison of age- and gender-specific *Campylobacter* enteritis rates in Germany in 2017.

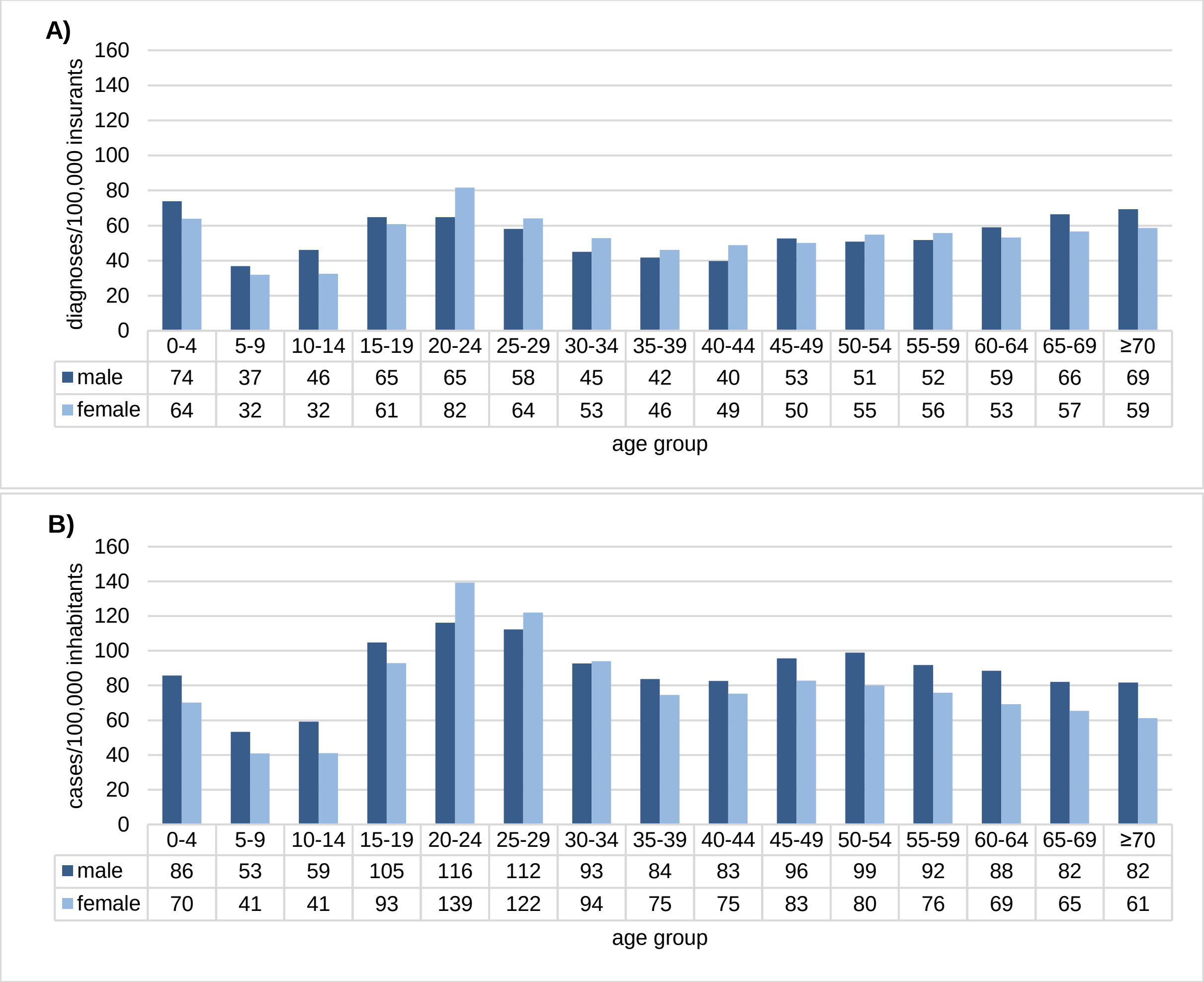

A)

	0-4	5-9	10-14	15-19	20-24	25-29	30-34	35-39	40-44	45-49	50-54	55-59	60-64	65-69	≥70
male	74	37	46	65	65	58	45	42	40	53	51	52	59	66	69
female	64	32	32	61	82	64	53	46	49	50	55	56	53	57	59

B)

	0-4	5-9	10-14	15-19	20-24	25-29	30-34	35-39	40-44	45-49	50-54	55-59	60-64	65-69	≥70
male	86	53	59	105	116	112	93	84	83	96	99	92	88	82	82
female	70	41	41	93	139	122	94	75	75	83	80	76	69	65	61

A) *Campylobacter* enteritis diagnoses of AOK insurants in 2017 per 100,000 insurants (number of AOK insurants according to [1] and personal communication from WIdO on the proportion of insurants <15 years). B) Officially reported *Campylobacter* enteritis cases per 100,000 inhabitants in Germany in 2017 according to national surveillance data [2].

Table A. Existing comorbidities in patients and controls during the first year of the individual study period according to the Elixhauser comorbidity index.

Comorbidities *[%]*	Study population			Subgroup		
	**Patients** *(n=9,945)*	**Controls**[a] *(n=29,835)*	**p value**	**Patients** *(n=4,036)*	**Controls**[b] *(n=9,350)*	**p value**
Congestive heart failure	11.42	6.65	<0.001	0.79	0.28	<0.001
Cardiac arrhythmias	15.96	9.67	<0.001	3.17	1.78	<0.001
Valvular disease	8.50	5.16	<0.001	1.41	0.74	<0.001
Pulmonary circulation disorders	2.72	1.45	<0.001	0.35	0.15	0.022
Peripheral vascular disorders	11.96	7.36	<0.001	1.14	0.41	<0.001
Hypertension	42.09	33.26	<0.001	8.87	4.62	<0.001
Paralysis	2.18	1.70	0.002	0.32	0.35	0.780
Other neurological disorders	5.60	3.50	<0.001	1.78	1.21	0.009
Chronic pulmonary disease	25.42	16.55	<0.001	12.31	8.59	<0.001
Diabetes, uncomplicated	18.05	12.57	<0.001	1.64	1.20	0.043
Diabetes, complicated	10.91	6.63	<0.001	0.40	0.17	0.014
Hypothyroidism	15.97	9.74	<0.001	4.73	2.98	<0.001
Renal failure	13.24	6.49	<0.001	1.16	0.52	<0.001
Liver disease	14.63	7.81	<0.001	3.94	1.65	<0.001
Peptic ulcer disease excluding bleeding	2.82	1.09	<0.001	0.47	0.18	0.003
AIDS/HIV	0.22	0.07	<0.001	0.02	0.00	0.128
Lymphoma	0.94	0.42	<0.001	0.17	0.05	0.033
Metastatic cancer	1.85	1.08	<0.001	0.32	0.12	0.010
Solid tumor without metastasis	9.55	6.41	<0.001	2.11	1.08	<0.001
Rheumatoid arthritis /collagen vascular diseases	7.04	3.93	<0.001	1.49	0.82	<0.001
Coagulopathy	4.34	2.06	<0.001	1.04	0.64	0.015
Obesity	20.67	13.10	<0.001	6.62	4.52	<0.001
Weight loss	3.95	1.29	<0.001	1.73	0.77	<0.001
Fluid and electrolyte disorders	33.85	3.50	<0.001	20.49	1.38	<0.001
Blood loss anemia	0.90	0.30	<0.001	0.35	0.07	<0.001
Deficiency anemia	7.63	3.90	<0.001	3.39	2.05	<0.001
Alcohol abuse	3.20	2.09	<0.001	0.94	0.76	0.282
Drug abuse	1.57	0.87	<0.001	0.79	0.47	0.023
Psychoses	1.61	1.06	<0.001	0.20	0.16	0.628
Depression	24.53	15.38	<0.001	9.42	6.11	<0.001

Comorbidities were assessed by applying the Elixhauser comorbidity index [3,4]. The Chi-square test was used to analyze statistically significant associations with the group assignment.

[a] Three controls per patient, matched by age, gender and state of residence.

[b] One to three controls per patient, matched by age, gender, state of residence and existing comorbidities (in terms of either the Elixhauser comorbidity index [3,4] or the pharmacy-based metric with the ATC algorithm [5], without *pain*).

Table B. Existing chronic conditions in patients and controls during the first year of the individual study period according to the pharmacy-based metric.

Chronic disease or medication *[%]*	Study population			Subgroup		
	**Patients** *(n=9,945)*	**Controls**[a] *(n=29,835)*	**p value**	**Patients** *(n=4,036)*	**Controls**[b] *(n=9,350)*	**p value**
Antiplatelet	2.46	1.29	<0.001	0.05	0.03	0.631
Anticoagulant	6.40	3.83	<0.001	0.84	0.75	0.571
Epilepsy	5.00	2.82	<0.001	0.12	0.14	0.826
Hypertension	8.17	4.85	<0.001	0.30	0.19	0.239
HIV	0.24	0.07	<0.001	0.02	0.00	0.128
Tuberculosis	0.10	0.02	0.001	0.00	0.00	.
Rheumatic conditions	11.86	5.79	<0.001	3.82	2.48	<0.001
Hyperlipidemia	17.05	12.26	<0.001	1.29	0.51	<0.001
Malignancies	0.58	0.25	<0.001	0.00	0.00	.
Parkinson’s disease	1.90	0.93	<0.001	0.12	0.05	0.171
Renal disease	0.65	0.20	<0.001	0.00	0.01	0.511
End stage renal disease	0.42	0.16	<0.001	0.00	0.00	.
Anti-arrhythmic	1.83	1.04	<0.001	0.00	0.00	.
Ischemic heart disease / Angina	2.13	1.13	<0.001	0.05	0.01	0.168
Congestive heart failure / Hypertension	32.58	25.21	<0.001	3.57	1.89	<0.001
Diabetes	11.59	7.53	<0.001	0.25	0.09	0.019
Glaucoma	2.07	1.69	0.014	0.12	0.12	0.924
Liver failure	0.50	0.23	<0.001	0.25	0.20	0.611
Acid peptic disease	39.37	17.57	<0.001	11.52	4.84	<0.001
Transplantation	0.98	0.23	<0.001	0.05	0.01	0.168
Respiratory illness, asthma	16.05	9.28	<0.001	7.78	5.53	<0.001
Thyroid disorders	15.20	10.70	<0.001	3.42	2.02	<0.001
Gout	6.49	3.87	<0.001	0.42	0.22	0.050
Inflammatory bowel disease, chronic	2.44	0.08	<0.001	1.02	0.01	<0.001
Pain and inflammation	37.96	30.43	<0.001	32.33	27.52	<0.001
Pain	4.83	2.32	<0.001	0.72	0.28	<0.001
Depression	11.71	6.93	<0.001	1.51	0.91	0.002
Psychotic illness	3.45	2.33	<0.001	0.30	0.17	0.143
Bipolar disorders	0.21	0.09	0.002	0.02	0.02	0.904
Anxiety and tension	3.19	1.64	<0.001	0.27	0.21	0.517
Hepatitis	0.09	0.02	<0.001	0.00	0.00	.
Ischemic heart disease / Hypertension	27.82	20.86	<0.001	2.80	1.26	<0.001

Chronic conditions were assessed by applying the pharmacy-based metric with the ATC algorithm [5]. The Chi-square test was used to analyze statistically significant associations with the group assignment.

[a] Three controls per patient, matched by age, gender and state of residence.

[b] One to three controls per patient, matched by age, gender, state of residence and existing comorbidities (in terms of either the Elixhauser comorbidity index [3,4] or the pharmacy-based metric with the ATC algorithm [5], without *pain*).

Table C. Association between the use of proton pump inhibitors and newly diagnosed *Campylobacter* enteritis.

Parameter	aOR	95 % confidence interval		p
**Use of proton pump inhibitors**[a] (reference: no use)	3.15	2.87	3.46	<0.001
**Male** (reference: female)	1.10	1.05	1.15	<0.001
**Age group** (reference: 45 to 64 years)				
<5 years	2.57	2.31	2.87	<0.001
5 to 14 years	2.43	2.18	2.71	<0.001
15 to 29 years	2.04	1.89	2.20	<0.001
30 to 44 years	1.61	1.49	1.74	<0.001
≥65 years	0.63	0.59	0.68	<0.001
**Comorbidities**[b] (reference: no comorbidities)				
one or two comorbidities	2.71	2.54	2.90	<0.001
three or more comorbidities	6.38	5.90	6.89	<0.001

Results of a multivariable logistic regression analysis with *Campylobacter* enteritis diagnosis (yes/no) as dependent variable, n=9,944 patients and n=29,832 controls included in the model. aOR: adjusted odds ratio.

[a] Prescriptions of proton pump inhibitors within four weeks prior to the individual study period according to the pharmacy dispensing date.

[b] Comorbidities were assessed by the Elixhauser comorbidity index [3,4].

Table D. Age- and gender-specific health care utilizations and work incapacities associated with diagnoses of *Campylobacter* enteritis over a 12-month period.

	Male						Female						Test for differences *[p value]*	
	**<5 y**	**5-14 y**	**15-29 y**	**30-44 y**	**45-64 y**	**≥65 y**	**<5 y**	**5-14 y**	**15-29 y**	**30-44 y**	**45-64 y**	**≥65 y**	**Age group**	**Gender**
**Moderate CE** *[n, %]*	234 (3.7)	225 (3.6)	583 (9.2)	485 (7.7)	982 (15.5)	563 (8.9)	192 (3.0)	175 (2.8)	648 (10.2)	567 (8.9)	957 (15.1)	724 (11.4)		
**Outpatient medical care** *[%]*														
GP	6.0	23.6	63.6	62.5	59.7	43.9	7.8	20.0	59.9	62.6	60.4	45.7	<0.001	0.470
pediatrician	83.3	65.3	4.6	0.0	0.0	0.0	80.7	69.1	3.2	0.4	0.0	0.0	<0.001	<0.001
internist	0.9	4.4	24.9	30.1	30.0	25.9	1.6	3.4	25.8	29.8	31.2	28.2	<0.001	0.106
other physicians	3.0	0.9	2.7	5.6	4.4	3.4	0.0	0.6	3.4	4.8	5.1	4.6	<0.001	0.493
provided in hospitals	0.0	0.4	0.7	0.0	0.1	0.2	0.0	0.0	0.3	0.2	0.0	0.0	0.042	0.173
**Prescribed medication**[a] *[%]*														
antibiotics	1.3	1.8	15.6	17.3	14.9	12.8	1.0	1.1	13.6	13.9	16.7	13.7	<0.001	0.857
analgesics/spasmolytics	32.1	13.3	10.3	13.4	18.1	21.5	33.3	14.3	12.7	15.2	20.5	27.9	<0.001	0.004
oral rehydration solution	14.1	9.3	0.3	0.0	0.1	0.0	17.7	7.4	0.3	0.0	0.0	0.0	<0.001	0.272
**Incapacities for work** *[%]*	-	-	18.2	20.2	19.8	0.4	-	-	17.7	18.9	18.5	0.0	<0.001	0.107
days [md (IQR, min-max)]			5 (3-9, 1-55)	7 (4-12, 1-247)	7 (4-13, 1-92)	12 (1-22, 1-22)			5 (2-10, 1-58)	9 (5-15, 1-164)	11 (5-17, 1-78)	-	<0.001[b]	0.004
**Severe CE** *[n, %]*	126 (3.5)	155 (4.3)	366 (10.1)	228 (6.3)	446 (12.4)	515 (14.3)	94 (2.6)	89 (2.5)	384 (10.6)	209 (5.8)	378 (10.5)	619 (17.2)		
**Outpatient medical care** *[%]*														
GP	0.8	3.2	13.1	20.6	15.9	10.1	1.1	3.4	15.4	17.7	13.0	9.4	<0.001	0.627
pediatrician	4.8	7.7	0.3	0.0	0.0	0.0	7.4	12.4	1.6	0.0	0.0	0.0	<0.001	0.378
internist	0.0	0.0	4.1	5.7	5.4	5.0	0.0	0.0	4.9	6.2	8.7	4.5	<0.001	0.159
other physicians	0.0	0.0	1.6	1.3	0.2	0.4	1.1	1.1	0.3	0.5	0.5	0.3	0.480	0.413
provided in hospitals	0.8	0.0	0.0	0.4	0.0	0.0	2.1	0.0	0.8	0.0	0.3	0.3	0.040	0.051
**Inpatient medical care**[c] *[%]*	100	100	100	100	100	100	100	100	100	100	100	100	.	.
stays [md (min-max)]	1 (1-2)	1 (1-1)	1 (1-2)	1 (1-2)	1 (1-2)	1 (1-2)	1 (1-1)	1 (1-1)	1 (1-2)	1 (1-2)	1 (1-2)	1 (1-2)	0.664	0.730
days per stay [md (IQR, min-max)]	4 (3-5, 1-12)	4 (3-4, 2-14)	4 (3-5, 1-37)	5 (4-6, 1-17)	5 (4-7, 2-53)	6 (5-8, 2-37)	4 (3-4, 2-9)	4 (3-5, 2-9)	4 (3-5, 1-23)	4 (3-6, 1-22)	5 (4-7, 2-46)	7 (5-9, 1-35)	<0.001[d]	<0.001
**Prescribed medication** *[%]*														
antibiotics	2.4	2.6	9.3	14.0	8.5	6.4	0.0	1.1	13.0	11.0	11.6	8.2	<0.001	0.072
analgesics/spasmolytics	44.4	11.6	12.0	19.7	25.8	24.7	35.1	16.9	16.9	21.5	28.8	32.8	<0.001	0.002
oral rehydration solution	18.3	1.9	0.0	0.0	0.0	0.0	18.1	3.4	0.0	0.0	0.0	0.0	<0.001	0.441
**Incapacities for work** *[%]*	-	-	53.0	75.0	57.2	0.4	-	-	52.6	66.5	51.9	0.2	<0.001	<0.001
days [md (IQR, min-max)]			2 (1-6, 1-28)	3 (1-8, 1-40)	4 (1-8, 1-64)	3 (1-5, 1-5)			2 (1-6, 1-21)	3 (1-7, 1-71)	4 (1-12, 1-207)	13	<0.001[e]	0.712

The percentage of patients utilizing the respective services or of patients who were incapacitated for work (%) and median (IQR, min-max) number of days or stays of utilizing patients are given. Existing differences or associations depending on age group and gender were examined using the Wilcoxon rank-sum, Kruskal-Wallis (followed by pairwise post-hoc Dunn's test) or Chi-square test, respectively. CE: *Campylobacter* enteritis, IQR: interquartile range, md: median, y: years.

[a] Considered to be CE-associated according to national medical treatment guidelines, as prescriptions of medication are not directly diagnosis-linked in the claims data.

[b] Pairwise comparisons using Dunn's test indicated statistically significant differences between 15-29 y and 30-44 y as well as 15-29 y and 45-64 y.

[c] Only hospitalizations with principal diagnoses were considered to be disease-associated and are displayed.

[d] Pairwise comparisons using Dunn's test indicated statistically significant differences between all groups except for <5 y and 5-14 y as well as 15-29 y and 30-44 y.

[e] Pairwise comparisons using Dunn's test indicated statistically significant differences between 15-29 y and 45-64 y as well as 30-44 y and 45-64 y.

Table E. Estimation of *Campylobacter* enteritis-related costs of outpatient medical care in the first year of the study period.

	**Part 1: Probability of non-zero costs** *(n=13,386)*			**Part 2: Non-zero cost estimation** *(n=11,639)*		
	**Coefficient**	**95 % CI**	**p**	**Coefficient**	**95 % CI**	**p**
**Male** (reference: female)	-0.88	-1.00 to -0.77	<0.001	-0.30	-0.34 to -0.27	<0.001
**Age group**						
<5 years	1.84	1.57 to 2.12	<0.001	0.14	0.07 to 0.20	<0.001
5 to 14 years	0.38	0.19 to 0.57	<0.001	-0.24	-0.30 to -0.17	<0.001
15 to 29 years	0.30	0.15 to 0.46	<0.001	-0.24	-0.29 to -0.18	<0.001
30 to 44 years	0.09	-0.09 to 0.26	0.324	-0.12	-0.18 to -0.06	<0.001
45 to 64 years	Reference			Reference		
≥65 years	0.28	-0.18 to 0.78	0.243	0.39	0.25 to 0.52	<0.001
***Campylobacter* enteritis** (CE)						
no CE	Reference			Reference		
moderate CE	5.81	4.68 to 7.61	<0.001	0.68	0.64 to 0.72	<0.001
severe CE	2.47	2.10 to 2.90	<0.001	0.36	0.31 to 0.42	<0.001
**Constant**	1.67	1.52 to 1.82	<0.001	5.14	5.09 to 5.19	<0.001
**Goodness-of-fit**						
McFadden's pseudo R^2	0.16					
adjusted R^2				0.13		

Results of a two-part regression model: i) probability of non-zero costs via logistic regression, ii) cost estimation via ordinary least squares regression of log-transformed non-zero costs; n=4,036 patients and n=9,350 controls, matched by age, gender, state of residence and comorbidities (in terms of either the Elixhauser comorbidity index [3,4] or the pharmacy-based metric with the ATC algorithm [5], without *pain*). CE: *Campylobacter* enteritis, CI: confidence interval.

Table F. Estimation of *Campylobacter* enteritis-related costs of prescribed medication in the first year of the study period.

	**Part 1: Probability of non-zero costs** *(n=13,386)*			**Part 2: Non-zero cost estimation** *(n=9,609)*		
	**Coefficient**	**95 % CI**	**p**	**Coefficient**	**95 % CI**	**p**
**Male** (reference: female)	-0.66	-0.74 to -0.58	<0.001	-0.14	-0.19 to -0.09	<0.001
**Age group**						
<5 years	2.31	2.08 to 2.56	<0.001	-0.08	-0.16 to 0.01	0.074
5 to 14 years	0.62	0.47 to 0.78	<0.001	-0.38	-0.47 to -0.30	<0.001
15 to 29 years	-0.16	-0.28 to -0.05	0.006	-0.39	-0.46 to -0.31	<0.001
30 to 44 years	-0.28	-0.41 to -0.15	<0.001	-0.41	-0.49 to -0.32	<0.001
45 to 64 years	Reference			Reference		
≥65 years	0.63	0.27 to 1.02	<0.001	0.96	0.78 to 1.13	<0.001
***Campylobacter* enteritis** (CE)						
no CE	Reference			Reference		
moderate CE	1.02	0.90 to 1.13	<0.001	0.28	0.23 to 0.34	<0.001
severe CE	0.87	0.72 to 1.03	<0.001	0.26	0.19 to 0.34	<0.001
**Constant**	0.91	0.79 to 1.03	<0.001	4.18	4.11 to 4.25	<0.001
**Goodness-of-fit**						
McFadden's pseudo R^2	0.10					
adjusted R^2				0.06		

Results of a two-part regression model: i) probability of non-zero costs via logistic regression, ii) cost estimation via ordinary least squares regression of log-transformed non-zero costs; n=4,036 patients and n=9,350 controls, matched by age, gender, state of residence and comorbidities (in terms of either the Elixhauser comorbidity index [3,4] or the pharmacy-based metric with the ATC algorithm [5], without *pain*). CE: *Campylobacter* enteritis, CI: confidence interval.

Table G. Mean age- and gender-specific costs of *Campylobacter* enteritis per patient over a 12-month period [in € (95 % confidence intervals)].

	**Male**						**Female**						**Test for differences** *[p value]*	
	**<5 y**	**5-14 y**	**15-29 y**	**30-44 y**	**45-64 y**	**≥65 y**	**<5 y**	**5-14 y**	**15-29 y**	**30-44 y**	**45-64 y**	**≥65 y**	**Age group**	**Gender**
**Moderate CE**														
**Direct costs**	168	137 (137-138)	139 (139-140)	159	187	292	215	163	167	189	221	342	<0.001[a]	<0.001
outpatient medical care	149	119	121	141	161	226	195	145	147	169	192	274	<0.001[b]	<0.001
outpatient medical care provided in hospitals	0	0 (0-1)	0 (0-1)	0	0	0	0	0	0	0	0	0	0.131	0.145
prescribed medication	19	17	18	18	27	65	21	18	20	20	29	69	<0.001[a]	<0.001
**Indirect costs**	0	0	381 (298-493)	546 (414-768)	559 (460-699)	11 (0-53)	0	0	311 (242-409)	499 (385-727)	497 (415-599)	0	<0.001[c]	0.329
**Total**	**168**	**137 (137-138)**	**521 (437-635)**	**703 (574-935)**	**746 (646-888)**	**303 (292-345)**	**215**	**163**	**478 (407-572)**	**687 (575-917)**	**717 (636-823)**	**342**	<0.001[d]	<0.001
**Severe CE**														
**Direct costs**	2,172 (2,088-2,273)	2,046 (1,965-2,188)	2,129 (2,008-2,532)	2,223 (2,098-2,616)	2,406 (2,305-2,573)	2,733 (2,603-2,940)	2,134 (2,052-2,216)	2,079 (1,999-2,275)	2,030 (1,985-2,090)	2,080 (2,013-2,172)	2,364 (2,276-2,549)	2,664 (2,571-2,803)	<0.001[e]	<0.001
outpatient medical care	71	63	64	76	87	120	90	72	73	85	97	136	<0.001[d]	<0.001
outpatient medical care provided in hospitals	1 (0-3)	0	0	0	0	0	0	0	0 (0-1)	0	0	0 (0-1)	0.400	0.543
inpatient medical care	2,082 (1,997-2,182)	1,967 (1,886-2,108)	2,049 (1,928-2,454)	2,131 (2,009-2,535)	2,295 (2,196-2,467)	2,553 (2,423-2,758)	2,025 (1,946-2,109)	1,990 (1,912-2,191)	1,939 (1,893-1,997)	1,977 (1,911-2,070)	2,240 (2,151-2,421)	2,465 (2,371-2,600)	<0.001[f]	0.766
prescribed medication	17	16	16	16	24	59	19	17	18	18	26	63	<0.001[d]	<0.001
**Indirect costs**	0	0	577 (482-688)	1,129 (931-1,370)	1,106 (931-1,333)	3 (0-14)	0	0	441 (368-520)	832 (648-1,140)	1,071 (820-1,544)	4 (0-13)	<0.001[g]	<0.001
**Total**	**2,172 (2,087-2,271)**	**2,046 (1,965-2,188)**	**2,706 (2,551-3,060)**	**3,351 (3,105-3,720)**	**3,512 (3,300-3,788)**	**2,736 (2,606-2,943)**	**2,134 (2,054-2,220)**	**2,079 (2,001-2,284)**	**2,471 (2,384-2,578)**	**2,912 (2,717-3,207)**	**3,434 (3,160-3,909)**	**2,668 (2,572-2,813)**	<0.001[h]	0.682

Costs of outpatient medical care provided in hospitals and inpatient medical care associated with diagnoses of *Campylobacter* enteritis (CE); costs of outpatient medical care and medication estimated as additional costs in comparison to controls; indirect costs correspond to work incapacities monetized with the average labor costs. Costs are given as mean and 95 % confidence intervals (based on 10,000 bootstrap samples). Existing differences between age groups and gender were examined using the Wilcoxon rank-sum or Kruskal-Wallis test (followed by pairwise post-hoc Dunn's test).

[a] Pairwise comparisons using Dunn's test indicated statistically significant differences between all groups.

[b] Pairwise comparisons using Dunn's test indicated statistically significant differences between all groups except for <5 y and 45-64 y.

[c] Pairwise comparisons using Dunn's test indicated statistically significant differences between all groups except for <5 y and 5-14 y, <5 and ≥65 y, 5-14 y and ≥65 y, 15-29 y and 30-44y, 15-29 y and 45-64 y as well as 30-44 y and 45-64 y.

[d] Pairwise comparisons using Dunn's test indicated statistically significant differences between all groups except for <5 y and 30-44 y.

[e] Pairwise comparisons using Dunn's test indicated statistically significant differences between all groups except for 5-14 y and 15-29 y as well as 5-14 y and 30-44 y.

[f] Pairwise comparisons using Dunn's test indicated statistically significant differences between all groups except for <5 y and ≥65 y, 5-14 y and 15-29 y, 5-14 y and 30-44 y as well as 15-29 y and 30-44 y.

[g] Pairwise comparisons using Dunn's test indicated statistically significant differences between all groups except for <5 y and 5-14 y, <5 y and ≥65 y, 5-14 y and ≥65 y as well as 15-29 y and 45-64 y.

[h] Pairwise comparisons using Dunn's test indicated statistically significant differences between all groups except for <5 y and 15-29 y as well as 30-44 y and 45-64 y.

Table H. Parameters and distribution functions used to extrapolate the costs of *Campylobacter* enteritis in Germany in 2017.

Model parameter	Distribution
***Campylobacter* enteritis (CE)**	
**Total number of CE cases**	
69,476 reported cases, age- and gender-specific case numbers [2]	no variation
**Number of severe CE cases**	
13,159 hospitalized cases, age- and gender-specific case numbers [6]	no variation
**CE mortality**	
8 deaths due to CE [7]; assumption: death occurs after severe CE [8,9]; full costs charged	no variation
**Costs of CE per person over 12 months**	
age- and gender-specific mean costs (Table G); reported CE cases with unknown age and/or gender were valued with the mean costs for moderate CE (Table 3)	PERT(lower CI, mean, upper CI of cost estimate)
**Adjustment for multiple CE infections per patient over 12 months**	
- moderate CE: 1.11 diagnoses per patient over 12 months	Uniform(1.00, 1.11)
- severe CE: 1.01 diagnoses per patient over 12 months	Uniform(1.00, 1.01)
**Sequelae**	
**Probability of developing reactive arthritis (REA)**	
0.12 % (Table 1);<br>sensitivity analysis: 1.7 % (95 % CI 0.5-5.8) [10]	no variation<br>PERT(0.5, 1.7, 5.8)
**Duration of REA**	
0.608 y [11]	Exponential(1/0.608)
**Probability of developing Guillain-Barré syndrome (GBS)**	
0.06 % (Table 1), age- and gender-specific distribution of cases according to [12];<br>sensitivity analysis: 0.07 % (95 % CI 0.03-0.15) [13]	no variation<br>PERT(0.03, 0.07, 0.15)
17 % of GBS cases are mild [14]	no variation
**Duration of GBS**	
- mild GBS for patients <15 y: 0.181 y [15]	Exponential(1/0.181)
- severe GBS for patients <15 y: 0.553 y (*C. jejuni* associated GBS cases [16])	Exponential(1/0.553)
- mild/severe GBS for patients ≥15 y: 0.530 y (average duration until healthy state is reached[a] [17])	Exponential(1/0.530)
- chronic GBS for patients ≥15 y: remaining age- and gender-specific life expectancy[b] [18]; 17-31 % of severe cases remain with permanent disability [9,14]	no variation;<br>Uniform(0.17, 0.31)
**GBS mortality**	
- age- and gender-specific mortality calculated as total deaths due to GBS [7] divided by total GBS cases estimated based on country-specific incidence rates [12,19]	PERT(deaths divided by lower CI, mean, upper CI of GBS incidence rate)
- deaths occur 33 days (0.09 y) after onset of severe GBS [17]; partial costs charged	Exponential(1/0.09)
**Probability of developing Crohn's disease (CD)**	
1.44 % (Table 1);<br>sensitivity analysis: 0.05-0.17 % [13]	no variation<br>Uniform(0.05, 0.17)
**Duration of CD**	
chronic [20]: remaining age- and gender-specific life expectancy[b] [18]	no variation

Table H. Parameters and distribution functions used to extrapolate the costs of *Campylobacter* enteritis in Germany in 2017 (continued).

Model parameter	Distribution
**Probability of developing ulcerative colitis (UC)**	
1.86 % (Table 1);<br>sensitivity analysis: 0.07-0.45 % [13]	no variation<br>Uniform(0.07, 0.45)
**Duration of UC**	
chronic [21]: remaining age- and gender-specific life expectancy[b] [18]	no variation
**Probability of developing irritable bowel syndrome (IBS)**	
3.39 % (Table 1);<br>sensitivity analysis: 4.0 % (95 % CI 1.4-10.9) [13]	no variation<br>PERT(1.4, 4.0, 10.9)
**Duration of IBS**	
5 y [22–24]	Exponential(1/5.0), maximal the remaining life expectancy[b] [18]
**Costs of REA / GBS / CD / UC / IBS per patient per 12 months**	
mean disease-related costs (Table 3)	PERT(lower CI, mean, upper CI of cost estimate)

CI: confidence interval, y: years.

[a] Reported proportions of healthy patients (F-score 0) were 0 % after 1 week, 1 % after 2 weeks, 5 % after 4 weeks, 28 % after 26 weeks, 35 % after 52 weeks [17]; a mean of 24 % are chronic cases [9,14]; therefore, the assumed disease duration of severe non-chronic cases was 1.5 weeks for 1 %, 3 weeks for 4 %, 15 weeks for 23 %, 39 weeks for 7 % and a maximum disease duration of 52 weeks for the remaining 41 %, resulting in an average disease duration of 27.6 weeks.

[b] The weighted age group-specific life expectancy was as follows [18]:

men: <5 y: 76.8 y, 5-14 y: 69.3 y, 15-29 y: 56.5 y, 30-44 y: 42.7 y, 45-64 y: 26.7 y, 65-74 y: 15.0 y, ≥75 y: 7.9 y;

women: <5 y: 81.5 y, 5-14 y: 74.1 y, 15-29 y: 61.2 y, 30-44 y: 47.1 y, 45-64 y: 30.5 y, 65-74 y: 17.7 y, ≥75 y: 8.8 y.

Table I. Extrapolation of cases and costs of *Campylobacter* enteritis in 2017 including sequelae in Germany.

	**Base case model**<br>*frequencies of sequelae as observed for AOK insurants*	**Sensitivity analysis**<br>*frequencies of sequelae according to published meta-analyses [13,10]*
**Cases**		
***Campylobacter* enteritis**	69,476	
Moderate cases	56,317	
Severe cases	13,159	
**Sequelae** [mean (95 % CI)]		
Reactive arthritis	85	1,193<br>(1,181-1,204)
Guillain-Barré syndrome	43	49<br>(48-49)
Crohn's disease	999	75<br>(74-75)
Ulcerative colitis	1,291	181<br>(179-182)
Irritable bowel syndrome	2,353	2,779<br>(2,758-2,801)
**Cost of illness** [mean (95 % CI), in € 1,000]		
***Campylobacter* enteritis**		
Direct costs	41,279<br>(41,270-41,287)	
Indirect costs	25,327<br>(25,308-25,347)	
Total costs	66,606<br>(66,581-66,630)	
**Sequelae**		
Reactive arthritis	45<br>(44-46)	631<br>(615-647)
Guillain-Barré syndrome	1,857<br>(1,843-1,870)	2,207<br>(2,184-2,230)
Crohn's disease	11,337<br>(11,279-11,395)	849<br>(842-856)
Ulcerative colitis	13,613<br>(13,566-13,660)	1,909<br>(1,892-1,927)
Irritable bowel syndrome	1,734<br>(1,708-1,760)	2,049<br>(2,012-2,086)
Total costs	28,585<br>(28,500-28,669)	7,646<br>(7,596-7,696)
**Total costs (discounted at 3 %)**	**95,191**<br>**(95,103-95,279)**	**74,252**<br>**(74,196-74,308)**
Total costs (undiscounted)	147,997<br>(147,767-148,227)	82,717<br>(82,615-82,818)
Total costs (discounted at 5 %)	83,102<br>(83,045-83,160)	72,051<br>(72,006-72,097)

Extrapolation is based on *Campylobacter* enteritis cases, hospitalizations and deaths as officially reported for Germany in 2017 [2,6,7]. Mean (95 % CI) number of cases and disease-related costs of *Campylobacter* enteritis and sequelae are presented (based on 10,000 iterations). CI: confidence interval.

References

1. Bundesministerium für Gesundheit. Statistik über Versicherte, gegliedert nach Status, Alter, Wohnort und Kassenart (Stichtag: 1. Juli 2017). Mitgliederstatistik KM6 [cited 4 Aug 2021]. Available from: https://www.bundesgesundheitsministerium.de/fileadmin/Dateien/3_Downloads/Statistiken/GKV/Mitglieder_Versicherte/KM6_2017.xlsx.
2. Robert Koch-Institut (RKI). SurvStat@RKI 2.0 [cited 4 May 2022]. Available from: https://survstat.rki.de.
3. Elixhauser A, Steiner C, Harris DR, Coffey RM. Comorbidity measures for use with administrative data. Med Care. 1998; 36:8–27. doi: 10.1097/00005650-199801000-00004.
4. Quan H, Sundararajan V, Halfon P, Fong A, Burnand B, Luthi J-C, et al. Coding algorithms for defining comorbidities in ICD-9-CM and ICD-10 administrative data. Med Care. 2005; 43:1130–9. doi: 10.1097/01.mlr.0000182534.19832.83.
5. Kuo RN, Dong Y-H, Liu J-P, Chang C-H, Shau W-Y, Lai M-S. Predicting healthcare utilization using a pharmacy-based metric with the WHO's Anatomic Therapeutic Chemical algorithm. Med Care. 2011; 49:1031–9. doi: 10.1097/MLR.0b013e31822ebe11.
6. Statistisches Bundesamt (Destatis). Tiefgegliederte Diagnosedaten der Krankenhauspatientinnen und -patienten 2017. 2018 [cited 4 Aug 2021]. Available from: https://www.statistischebibliothek.de/mir/servlets/MCRFileNodeServlet/DEHeft_derivate_00056617/5231301177015.xlsx.
7. Statistisches Bundesamt (Destatis). Gesundheit. Ergebnisse der Todesursachenstatistik für Deutschland 2017. 2019. Available from: https://www.destatis.de/DE/Themen/Gesellschaft-Umwelt/Gesundheit/Todesursachen/Publikationen/Downloads-Todesursachen/todesursachenstatistik-5232101177015.xlsx?__blob=publicationFile.
8. Lackner J, Weiss M, Müller-Graf C, Greiner M. The disease burden associated with Campylobacter spp. in Germany, 2014. PLoS One. 2019; 14:e0216867. doi: 10.1371/journal.pone.0216867.
9. European Centre for Disease Prevention and Control (ECDC). ECDC BCoDE toolkit [software application]. Version 2.0.0. 2020 [cited 9 Nov 2020]. Available from: https://www.ecdc.europa.eu/en/publications-data/toolkit-application-calculate-dalys.
10. Pogreba-Brown K, Austhof E, Tang X, Trejo MJ, Owusu-Dommey A, Boyd K, et al. Enteric Pathogens and Reactive Arthritis: Systematic Review and Meta-Analyses of Pathogen-Associated Reactive Arthritis. Foodborne Pathog Dis. 2021; 18:627–39. doi: 10.1089/fpd.2020.2910.
11. Mangen M, Havelaar AH, Wit GA de. Campylobacteriosis and sequelae in the Netherlands. Estimating the disease burden and the cost-of illness. National Institute for Public Health and the Environment (RIVM); 2004. Available from: https://www.rivm.nl/bibliotheek/rapporten/250911004.pdf.
12. Hense S, Schink T, Kreisel SH, Marcelon L, Simondon F, Tahden M, et al. Estimation of background incidence rates of Guillain-Barré syndrome in Germany - a retrospective cohort study with electronic healthcare data. Neuroepidemiology. 2014; 43:244–52. doi: 10.1159/000369344.
13. Keithlin J, Sargeant J, Thomas MK, Fazil A. Systematic review and meta-analysis of the proportion of Campylobacter cases that develop chronic sequelae. BMC Public Health. 2014; 14:1203. doi: 10.1186/1471-2458-14-1203.
14. Havelaar AH, Wit MA de, van Koningsveld R, van Kempen E. Health burden in the Netherlands due to infection with thermophilic Campylobacter spp. Epidemiol Infect. 2000; 125:505–22. doi: 10.1017/s0950268800004933.
15. Korinthenberg R, Mönting JS. Natural history and treatment effects in Guillain-Barré syndrome: a multicentre study. Archives of Disease in Childhood. 1996; 74:281–7. doi: 10.1136/adc.74.4.281.
16. Schessl J, Luther B, Kirschner J, Mauff G, Korinthenberg R. Infections and vaccinations preceding childhood Guillain-Barré syndrome: a prospective study. Eur J Pediatr. 2006; 165:605–12. doi: 10.1007/s00431-006-0140-1.
17. Doets AY, Verboon C, van den Berg B, Harbo T, Cornblath DR, Willison HJ, et al. Regional variation of Guillain-Barré syndrome. Brain. 2018; 141:2866–77. doi: 10.1093/brain/awy232.
18. Statistisches Bundesamt (Destatis). Sterbetafeln 2016/2018. Ergebnisse aus der laufenden Berechnung von Periodensterbetafeln für Deutschland und die Bundesländer. 2019.
19. Statistisches Bundesamt (Destatis). Bevölkerung: Bundesländer, Stichtag, Geschlecht, Altersjahre. Tabelle 12411-0013 [cited 8 Dec 2021]. Available from: https://www-genesis.destatis.de/genesis/online?operation=statistic&levelindex=0&levelid=1584541234414&code=12411.
20. Sturm A, Atreya R, Bettenworth D, Bokemeyer B, Dignaß A, Ehehalt R, et al. Aktualisierte S3-Leitlinie "Diagnostik und Therapie des Morbus Crohn". Deutsche Gesellschaft für Gastroenterologie, Verdauungs- und Stoffwechselkrankheiten (DGVS), editor. 2021. Available from: https://www.awmf.org/uploads/tx_szleitlinien/021-004l_S3_Morbus_Crohn_Diagnostik_Therapie_2021-08.pdf.
21. Kucharzik T, Dignass AU, Atreya R, Bokemeyer B, Esters P, Herrlinger K, et al. Aktualisierte S3-Leitlinie Colitis ulcerosa – Living Guideline. Z Gastroenterol. 2020; 58:e241-e326. doi: 10.1055/a-1296-3444.

22. Youn YH, Kim HC, Lim HC, Park JJ, Kim J-H, Park H. Long-term Clinical Course of Post-infectious Irritable Bowel Syndrome After Shigellosis: A 10-year Follow-up Study. J Neurogastroenterol Motil. 2016; 22:490–6. doi: 10.5056/jnm15157.
23. Marshall JK, Thabane M, Garg AX, Clark WF, Moayyedi P, Collins SM. Eight year prognosis of postinfectious irritable bowel syndrome following waterborne bacterial dysentery. Gut. 2010; 59:605–11. doi: 10.1136/gut.2009.202234.
24. Neal KR, Barker L, Spiller RC. Prognosis in post-infective irritable bowel syndrome: a six year follow up study. Gut. 2002; 51:410–3. doi: 10.1136/gut.51.3.410.
